# Self‐Reconstructed Spinel Surface Structure Enabling the Long‐Term Stable Hydrogen Evolution Reaction/Oxygen Evolution Reaction Efficiency of FeCoNiRu High‐Entropy Alloyed Electrocatalyst

**DOI:** 10.1002/advs.202300094

**Published:** 2023-03-22

**Authors:** Kang Huang, Jiuyang Xia, Yu Lu, Bowei Zhang, Wencong Shi, Xun Cao, Xinyue Zhang, Lilia M. Woods, Changcun Han, Chunjin Chen, Tian Wang, Junsheng Wu, Yizhong Huang

**Affiliations:** ^1^ Institute for Advanced Materials and Technology University of Science and Technology Beijing Beijing 100083 P. R. China; ^2^ School of Materials Science and Engineering Nanyang Technological University 50 Nanyang Avenue Singapore 639798 Singapore; ^3^ School of Biological Sciences Nanyang Technological University 50 Nanyang Avenue Singapore 639798 Singapore; ^4^ Department of Physics University of South Florida Tampa FL 33620 USA; ^5^ College of Science Hubei University of Technology Wuhan 430068 P. R. China; ^6^ Institute of Metal Research Chinese Academy of Sciences Shenyang 110016 P. R. China; ^7^ Department of Chemistry National University of Singapore 3 Science Drive 3 Singapore 117543 Singapore

**Keywords:** atomic lattice hollow sites, high‐entropy alloys, overall water splitting, spinel oxide, surface self‐reconstruction

## Abstract

High catalytic efficiency and long‐term stability are two main components for the performance assessment of an electrocatalyst. Previous attention has been paid more to efficiency other than stability. The present work is focused on the study of the stability processed on the FeCoNiRu high‐entropy alloy (HEA) in correlation with its catalytic efficiency. This catalyst has demonstrated not only performing the simultaneous hydrogen evolution reaction (HER) and oxygen evolution reaction (OER) with high efficiency but also sustaining long‐term stability upon HER and OER. The study reveals that the outstanding stability is attributed to the spinel oxide surface layer developed during evolution reactions. The spinel structure preserves the active sites that are inherited from the HEA's intrinsic structure. This work will provide an insightful direction/pathway for the design and manufacturing activities of other metallic electrocatalysts and a benchmark for the assessment of their efficiency–stability relationship.

## Graphical abstract

The stability of electrocatalysts is correlated with their surface structures that are self‐reconstructed responding to the hydrogen evolution reaction (HER) or oxygen evolution reaction (OER). The spinel oxide, inherited from the original high‐entropy alloy catalyst and developed over the surface of FeCoNiRu when subject to the polarization, provides the hollow active sites enabling the long‐term stabilities of HER and OER.

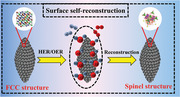

## 1 Introduction

The electrocatalysis is known to be a process that occurs in the interface between the surface of electrocatalysts and various electrolytes.^[^ ^1^ ^]^ The vast majority of research efforts have addressed the synthesis of new and cost‐effective catalytic materials to pursue high catalytic performance over the past decades.^[^ ^2^, ^3^, ^4^, ^5^ ^]^ However, long‐term stability, an essential characteristic of electrocatalysts, has not been in‐depth studied and understood so far.^[^ ^6^, ^7^, ^8^ ^]^ The poor stability has substantially restrained the practical electrocatalytic applications.^[^ ^9^ ^]^ Water splitting, for instance, involves two half‐reactions, that is, hydrogen evolution reaction (HER) happening on the cathode and oxygen evolution reaction (OER) on the anode,^[^ ^10^, ^11^ ^]^ and has been extensively studied with the assistance of electrocatalysts.^[^ ^12^, ^13^, ^14^, ^15^ ^]^ Subject to the applied potential and aggressive environments, the redox reactions occurring on both electrodes require their strong durability for a long‐term run. The execution of catalytic activities facilitated by catalysts is well understood through the kinetic process on the surface of electrodes as determined by active sites.^[^ ^16^, ^17^ ^]^ However, the fundamental catalytic mechanism by which the electrodes are maintained in a stable state during the electrochemical reaction in the electrolytic cell is still unclear due to the lack of investigation.^[^ ^18^, ^19^, ^20^ ^]^ This is result of the difficulty in the simultaneous inspection of the self‐reconstruction over the surface of electrodes when the stability test is in progress.^[^ ^21^, ^22^ ^]^

Alloys are one type among the vast majority of catalysts that have been widely used in the electrocatalysis of water splitting. It is a process that occurs through surface metal atoms dissolution, in situ oxidation of metal atoms, dissolution‐redeposition procedure, or their combination. The products over the surface alloys developed during the self‐reconstruction process are highly dependent on the specific substrate and relevant environments and can increase or decrease the catalytic activity of alloys.^[^ ^23^, ^24^, ^25^, ^26^, ^27^, ^28^, ^29^ ^]^ In addition, self‐reconstruction must consider other factors such as the morphology, composition, and lattice phase structure of alloys. So there is no single self‐reconstruction mechanism is applicable to the diverse alloys. This motivates us to explore why FeCoNiRu high‐entropy alloy (HEA) remains equally high efficiency even though its surface is covered with a layer of oxide formed by self‐reconstruction.

HEAs, have attracted great interest as electrocatalysts for water splitting than binary and ternary alloys in recent years due to their huge potential in composition and electron structure adjustment. However, the differences in chemical and physical properties of each element lead to great difficulties in synthesizing single‐phase HEA. Metal‐organic frameworks (MOFs) template method is proved to be a simple and effective approach to synthesizing HEA nanoparticles for the first time in our previous work.^[^ ^14^ ^]^ Therefore, in the present work, FeCoNiRu HEA electrocatalyst derived from high‐entropy metal‐organic frameworks (HEMOFs) precursor, is prepared. The carbon skeleton derived from MOF precursor with a porous structure and sufficient channels allows the fast transfer of reaction species, mitigates the growth of nanoparticles, and avoids their aggregation. It also enables excellent structural and compositional stability against the HER and OER. Meanwhile, due to the huge difference in electronegativity between Ru and other transition metals, the addition of Ru will give rise to the numerous active sites over the surface of HEA which are beneficial for both HER and OER. This renders the alloy that can act as both anode and cathode at the same time in an overall electrolytic cell. As a result, the stabilities of HER and OER can be evaluated simultaneously at identical conditions.

## 2 Results and Discussion

### 2.1 Characterization of the FeCoNiRu‐450 Electrocatalyst

**Figure 1:**
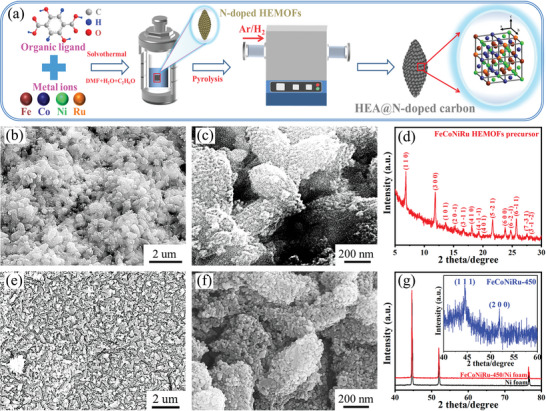
Morphological and phase structure characterization. a) Schematic of the synthesis of FeCoNiRu HEAs composites. b) Low and c) high magnification FESEM images and d) XRD spectrum of HEMOFs precursor. e) Low and f) high magnification FESEM images and g) XRD spectrum of FeCoNiRu‐450.

The synthetic process of FeCoNiRu‐450 composites is schematically illustrated in **Figure** **1**a. The FeCoNiRu HEMOFs overall precursor was viewed to be uniformly assembled as shown in Figure 1b, a secondary electron image taken in a scanning electron microscope (SEM) at low magnification. It appears a rice shape with a rough surface in the magnified image (Figure 1c) and was determined as a single crystal structure by X‐ray diffraction (XRD) (Figure 1d). After the pyrolysis, FeCoNiRu HEMOFs were completely decomposed into the carbon skeleton without collapsing and agglomerating, as indicated in Figure 1e,f. The shape of HEMOF remains intact but the surface is decorated by considerable tiny nanoparticles over the skeleton (Figure 1f), which are estimated to be under 10 nanometers in size. This is demonstrated by the fairly broad face‐centered cubic (FCC) XRD peaks (inset) in Figure 1g. The content of each element in FeCoNiRu‐450 was determined from the measurement of an inductively coupled plasma mass‐spectrometry (ICP‐MS, Figure S5, Supporting Information). The result suggests that the atomic percentage of each element in FeCoNiRu‐450 is between 5% and 35%, in consistence with the designed HEAs.^[^ ^30^ ^]^

**Figure 2:**
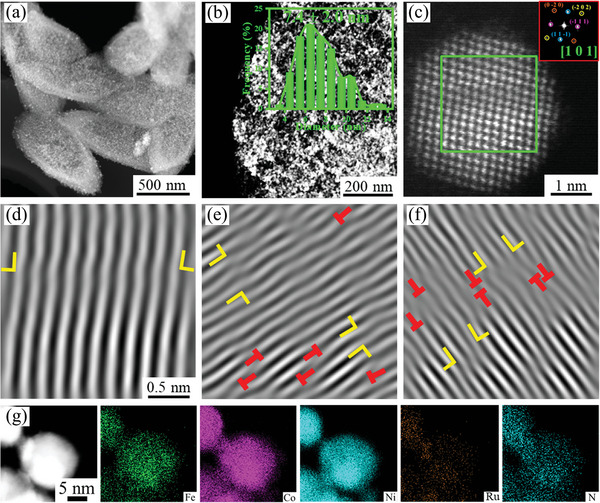
HAADF‐STEM and elemental analysis of FeCoNiRu‐450. a) Low magnification STEM image, b) high magnification STEM image (inset: size distribution), c) high‐resolution STEM image, d–f) the IFFT images transformed along (0 −2 2), (−2 2 0), and (−2 0 2), g) elemental maps.

Further analysis of microstructures of FeCoNiRu‐450 using high‐angle annual dark‐field scanning transmission electron microscopy (HAADF‐STEM) proves the uniform dispersion of nanoparticles on the carbon skeleton (**Figure** **2**a) with the average size of 7.4 ± 2.0 nm estimated from their size distribution (inset in Figure 2b). The atomic STEM image confirms the FCC nanocrystalline structure of the FeCoNiRu‐450 (Figure 2c). Figure 2d–f, is the inverse fast Fourier transform (IFFT) images performed from (1 1 1), (2 0 0), and (2 2 0) lattice planes, which enable revealing the lattice defects such as stacking faults and dislocations. The yellow and red marks indicate the stacking faults and dislocations, respectively. These stacking faults and dislocations can accelerate the electrochemical processes by declining the reaction activation energy. The elemental mapping was examined using STEM energy‐dispersive X‐ray mapping. All the elements are uniformly distributed within nanoparticles without elemental segregation and/or phase separation (Figure 2g). Importantly, N atoms are also successfully doped into carbon frameworks leading to additional defect active sites and improvement of the electrical conductivity of the electrocatalyst.^[^ ^31^ ^]^

The additional characterization of FeCoNiRu‐450 was carried out using BET (Figure S6, Supporting Information), Raman spectroscopy (Figure S7, Supporting Information), and X‐ray photoelectron spectroscopy (XPS) (Figure S8, Supporting Information). These results are all presented in Supporting Information.

### 2.2 Hydrogen Evolution Reaction Evaluation of the FeCoNiRu‐450 Electrocatalyst

**Figure 3:**
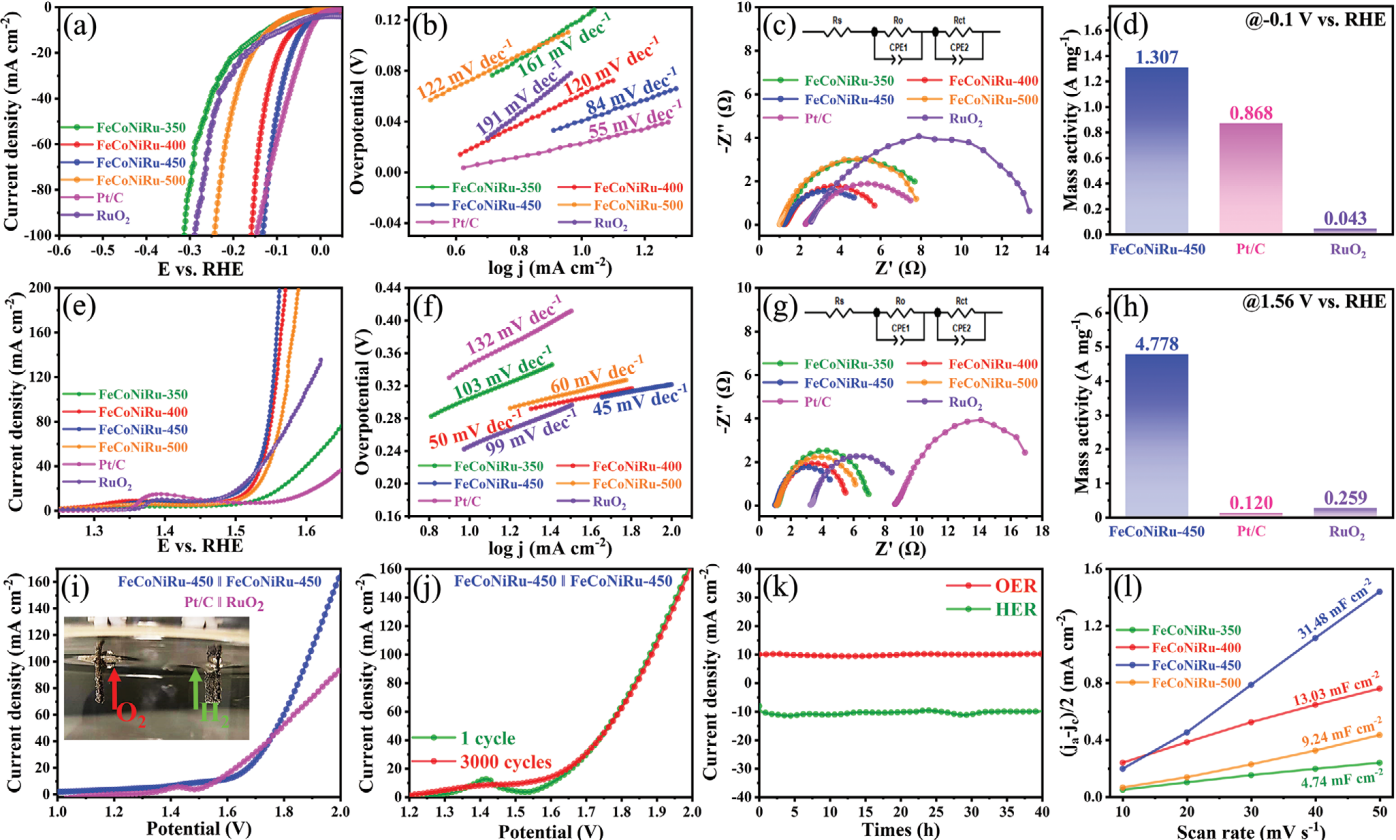
Electrocatalytic performance evaluation of FeCoNiRu electrocatalysts. a) LSV, b) Tafel, c) EIS, d) mass activity for HER, e) LSV, f) Tafel, g) EIS, h) mass activity for OER, i) overall water splitting without iR compensation, j) LSV of FeCoNiRu‐450‖FeCoNiRu‐450 before and after 3000 cycles, k) current density‐time curve, and l) *C*_dl_ values.

**Figure** **3**a shows the electrocatalytic HER performance of as‐prepared electrocatalysts in which FeCoNiRu‐450 exhibits the best HER catalytic activity comparable to commercial Pt/C, which requires an overpotential of 40 mV to achieve a current density of 10 mA cm^−2^, smaller than FeCoNiRu‐350 (122 mV), FeCoNiRu‐400 (61 mV), FeCoNiRu‐500 (117 mV), and the commercial RuO_2_ (87 mV). The electrocatalytic HER performance of FeCoNiRu‐450 is also superior to that of the recently reported HEAs and noble metal‐based HER electrocatalysts counterparts. The comparison of them is listed in Table S1, Supporting Information. The smaller Tafel slope of FeCoNiRu‐450 (84 mV dec^−1^) than FeCoNiRu‐350 (161 mV dec^−1^), FeCoNiRu‐400 (120 mV dec^−1^), FeCoNiRu‐500 (122 mV dec^−1^), and the commercial RuO_2_ (191 mV dec^−1^), indicates it's most favorable catalytic HER reaction kinetics closing to the commercial Pt/C (Figure 3b). Nyquist curves from as‐prepared electrocatalysts were measured using the electrochemical impedance spectroscopy (EIS) technique to further prove the reaction kinetics of the electrode.^[^ ^32^, ^33^ ^]^ As shown in Figure 3c, FeCoNiRu‐450 shows the smallest diameter of the semi‐arc of charge transfer resistance compared to others, suggesting its excellent charge transfer capacity (Tables S2 and S3, Supporting Information). The normalized mass activity of precious metals is an essential reference to practical applications of electrocatalysts. FeCoNiRu‐450 and the commercial noble metal‐based electrocatalysts (Pt/C and RuO_2_) were both estimated at *η* = 100 mV with the results presented in Figure 3d, where FeCoNiRu‐450 supplies the higher mass activity than the commercial Pt/C and 30 times more than that of the commercial RuO_2_. It is a potential HER electrocatalyst that could replace the commercial Pt/C for practical applications. In addition, electrochemically active surface area (ECSA) reflects the intrinsic HER catalytic activity of the as‐prepared electrocatalysts, which can be determined by the double‐layer capacitance (*C*_dl_). As shown in Figure 3l, the FeCoNiRu‐450 electrocatalyst provides the highest *C*_dl_ value (31.48 mF cm^−2^) compared to FeCoNiRu‐350 (4.74 mF cm^−2^), FeCoNiRu‐400 (13.03 mF cm^−2^), and FeCoNiRu‐500 (9.24 mF cm^−2^), demonstrating that the FeCoNiRu‐450 electrocatalyst affords the most catalytic active sites. To further clarify the intrinsic catalytic activity of electrocatalysts, turnover frequency (TOF), as another powerful parameter has been calculated. FeCoNiRu‐450 shows a TOF value of 0.046 S^−1^ at *η* = 100 mV, which is comparable to the commercial Pt/C electrocatalyst and twice more than the commercial RuO_2_ electrocatalyst (Table S4, Supporting Information). It further proves the outstanding HER performance of the FeCoNiRu‐450 electrocatalyst. Besides, the long‐term stability of HER electrocatalysts is considered a more important factor than the activity of catalytic for practical applications. The remarkable long‐term stability of the FeCoNiRu‐450 electrocatalyst was evaluated by chronoamperometric technique under reaction conditions (Figure 3k). The current density is constantly maintained at 10 mA cm^−2^ after 40 h. It is worth noting that the HER performance of the FeCoNiRu‐450 electrocatalyst is also better than unary Co, binary FeCo, ternary FeCoNi, FeCoRu, FeNiRu, and CoNiRu electrocatalysts with the measured results shown in Figure S16 and Table S5, Supporting Information.

### 2.3 Oxygen Evolution Reaction Evaluation of the FeCoNiRu‐450 Electrocatalyst

Parallel to the HER, the OER performance of the as‐prepared electrocatalyst has also been evaluated. Linear sweep voltammetry (LSV) curves shown in Figure 3e demonstrate that FeCoNiRu‐450 delivers the best OER performance with the minimum overpotential value of only 243 mV required to reach a current density of 10 mA cm^−2^ in comparison with FeCoNiRu‐350 (304 mV), FeCoNiRu‐400 (269 mV), FeCoNiRu‐500 (276 mV), commercial RuO_2_ (258 mV), and commercial Pt/C (346 mV). The electrocatalytic OER performance of FeCoNiRu‐450 is also better than that of the recently reported HEAs and precious‐metal‐based OER electrocatalyst counterparts (Table S7, Supporting Information). FeCoNiRu‐450 also exhibits the fastest catalytic OER kinetics and mechanism over others. Figure 3f shows that the FeCoNiRu‐450 electrocatalyst possesses a Tafel slope of 45 mV dec^−1^, which is lowest amongst FeCoNiRu‐350 (103 mV dec^−1^), FeCoNiRu‐400 (50 mV dec^−1^), FeCoNiRu‐500 (60 mV dec^−1^), commercial RuO_2_ (79 mV dec^−1^), and commercial Pt/C (132 mV dec^−1^), indicating its fastest electrode reaction kinetics. It can be further proven by Nyquist curves as shown in Figure 3g. The smallest diameter of the semi‐arc of charge transfer resistance over others suggests the superb charge transfer capacity (Figure 3g, Tables S8 and S9, Supporting Information). The mass activity of FeCoNiRu‐450 and commercial noble metal‐based (Pt/C and RuO_2_) is both estimated at *η* = 330 mV with the results shown in Figure 3h, where the FeCoNiRu‐450 electrocatalyst supplies the highest mass activity, 18 times more than the commercial RuO_2_ and ≈40 times more than the commercial Pt/C. Meanwhile, the TOF value of FeCoNiRu‐450 was calculated to be 0.084 S^−1^ based on the same overpotential, which is higher than the commercial RuO_2_ and 28 times larger than the commercial Pt/C (Table S4, Supporting Information) implying its superior OER performance. The long‐term stability of the OER electrodes is also considered a key factor in practice. The stability of the FeCoNiRu‐450 electrocatalyst is evaluated by the chronoamperometric technique convincing of its remarkable long‐term stability under reaction conditions (Figure 3k). The current density is constantly maintained at 10 mA cm^−2^ after 40 h. Besides, the OER performance of FeCoNiRu‐450 is also better than unary Co, binary FeCo, ternary FeCoNi, FeCoRu, FeNiRu, and CoNiRu as shown in Figure S18 and Table S10, Supporting Information.

### 2.4 Overall Water Splitting Evaluation of the FeCoNiRu‐450 Electrocatalyst

FeCoNiRu‐450 combined with the equally outstanding performances in both catalytic efficiency and stability of HER and OER can act as a bifunctional electrocatalyst in an overall cell for water splitting. The overall splitting activity of FeCoNiRu‐450 was examined in 1 m KOH in a two‐electrode configuration, where FeCoNiRu‐450 serves both cathodes for HER and anode for OER without iR compensation (inset of Figure 3i). For comparison, a similar two‐electrode cell consisting of commercial Pt/C as the cathode for the HER and commercial RuO_2_ as the anode for the OER was also constructed. As shown in Figure 3i, FeCoNiRu‐450 prevails better overall water splitting performance with a cell voltage of 1.563 V to attain the current density of 10 mA cm^−2^, than commercial noble metal formed two‐electrode system (1.565 V). As a consequence, the higher current density at high voltage provided by the FeCoNiRu‐450 catalytic system than the commercial noble metal catalytic system, determines the better catalytic kinetics of FeCoNiRu‐450 than the commercial noble metals toward overall water splitting. In addition, FeCoNiRu‐450 also shows prominent overall water‐splitting stability as proven by the almost overlap of the *I*–*V* curve before and after 3000 cycles (Figure 3j).

### 2.5 Density Functional Theory Calculation

**Figure 4:**
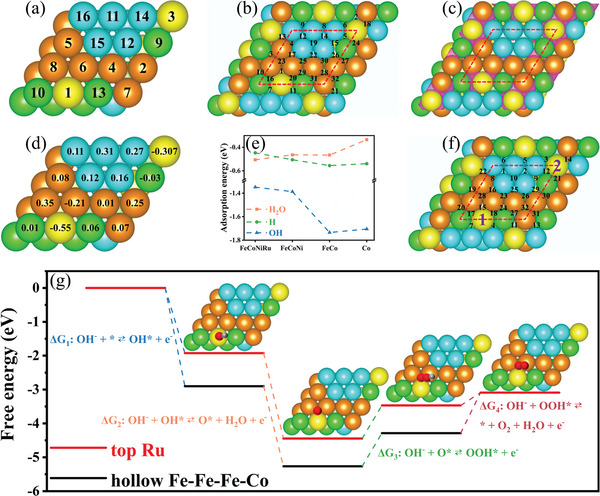
DFT calculations. a) The top sites for H_2_O adsorption. b) The hollow sites for H^+^ adsorption. c) Append with the CDD plot for the alloy model. The isovalue was set 0.012 eV bohr^−3^. The pink color indicates the accumulation of electrons. d) Bader analysis. e) H_2_O, H, and OH adsorption free energy of FeCoNiRu HEA, ternary FeCoNi alloy, binary FeCo alloy, and unary Co. f) The Ru top sites and hollow sites for OH^−^ adsorption. g) Schematic of the OER mechanism on the Ru top site 1 and hollow site 32 (Fe‐Fe‐Fe). Yellow is Ru, cyan is Ni, green is Co, brown is Fe, red is O, and grey is H.

To reveal the excellent overall water splitting performance of FeCoNiRu, density functional theory (DFT) calculations based on the first principal theory were performed in which the slab model of FCC FeCoNiRu was constructed and the Monte Carlo method was used to optimize the randomly distributed elements. The {1 1 1} lattice facet is selected as the exposed surface since it is most stable over the rest lattice facets (Figure S20, Supporting Information). All the possible adsorption positions (i.e., hollow, bridge, and top sites) of H_2_O, H^+^, and OH^−^ were calculated. The results show that the top sites of metal are the places for the stable adsorption of H_2_O molecules. The H^+^ adsorption is only stable on the surface hollow sites. In contrast, the OH^−^ adsorption is stable on the top sites of Ru besides the hollow sites. For HER in alkaline conditions, the adsorption of H_2_O is considered a critical process for the catalysis of water. Based on the calculation, the sequential stability order of H_2_O adsorption is numbered in **Figure** **4**a with the most stable top site of Ru (Table S12, Supporting Information). It is demonstrated that Ru enables the improvement of the electrocatalytic HER performance of HEAs. In addition, the desorption of H_2_ is the last process of HER. The lower adsorption‐free energy of H^+^ leads to better electrocatalytic HER performance due to stronger H_2_ desorption. The hollow sites marked in Figure 4b require the lowest energy for H_2_ molecules desorption, which is positioned near the Ru atom (see Table S13, Supporting Information, for more details). It is further proved that Ru can enhance the electrocatalytic HER performance of HEAs. To in‐depth understand the intrinsic mechanism that the Ru element promotes the HER, the charge density differences (CDD) of the slab model were calculated. The CDD can predict the direction of electrons of each compositional element in HEAs during their formation process. As shown in Figure 4c, Ru atoms tend to donate valence electrons to the surrounding hollow sites due to the large difference in electronegativity between Ru and other transition metals. For HER, H_2_O molecules with two pairs of isolated electrons each intend to adsorb onto the surface sites of elements that exhibit more empty orbits. Therefore, the Ru top site is the preferential place for H_2_O adsorption. Meanwhile, valence electrons accumulate at the hollow sites near Ru atoms and their filling into the antibonding orbitals of the adsorbate will weaken the coupling strength between the adsorbate and alloy surface.^[^ ^34^ ^]^ In addition, to determine the electron gains and losses of each surface atom (Figure 4d, a positive number means the gain of electrons, and negative number the loss of electrons), the worse charge of the surface atoms was calculated.^[^ ^35^ ^]^ The results show that the electrons transfer from the atom of Ru to the transition metal atoms in close of Ru. These transition metal atoms gain additional electrons resulting in the electron‐enriched hollow sites that weaken the adsorption energy of H_2_. The Ru top sites are the best position for H_2_O adsorption whilst the hollow sites close to Ru are the preferential positions for H_2_ desorption. Apart from the FeCoNiRu system, the H_2_O/H‐adsorption free energy of unary Co, binary FeCo, and ternary FeCoNi alloys were also calculated. As shown in Figure 4e, the FeCoNiRu HEA presents the strongest H_2_O‐adsorption free energy and lowest H‐adsorption free energy over others, proving that the FeCoNiRu HEA wins the best HER.

Hence, the addition of the Ru element enables significant HER enhancement, in agreement with the experimental results above. In contrast, the OER is a more complex reaction process that involves a four‐electron transfer in the course of OH^−^ adsorption and O_2_ desorption. Since OH^−^ can be stably adsorbed on both Ru top sites and hollow sites, the adsorption energies of OH^−^ over these sites were also calculated. Two Ru top sites with the lowest OH^−^ adsorption‐free energy are marked by purple numbers in Figure 4f with their corresponding adsorption‐free energies presented in Table S14, Supporting Information. Due to the consistent changes of the reaction enthalpy across the whole OER pathway, the lower OH^−^ adsorption‐free energy is associated with the lower O_2_ desorption energy, that is, better electrocatalytic OER performance. The free energy of the entire OER reaction path for the Ru top site (the lowest OH^−^ adsorption‐free energy) and the hollow site 32 (the highest OH^−^ adsorption energy) were further calculated. The free energy of each OER step at the Ru top site rises up compared to the hollow site 32 (Figure 4g) demonstrating that the incorporation of Ru is favorable for the OER of HEAs. Besides, the calculations of the OH‐adsorption free energy of unary Co, binary FeCo, and ternary FeCoNi alloys show that the FeCoNiRu HEA exhibits the lowest OH‐adsorption free energy over others (Figure 4e). This is additional evidence to prove that the Ru accommodated in HEAs can further enhance the OER performance in good alignment with the experimental results above.

### 2.6 Surface Structures of the FeCoNiRu‐450 Electrocatalyst upon the Long‐Term Hydrogen Evolution Reaction Stability Test

**Figure 5:**
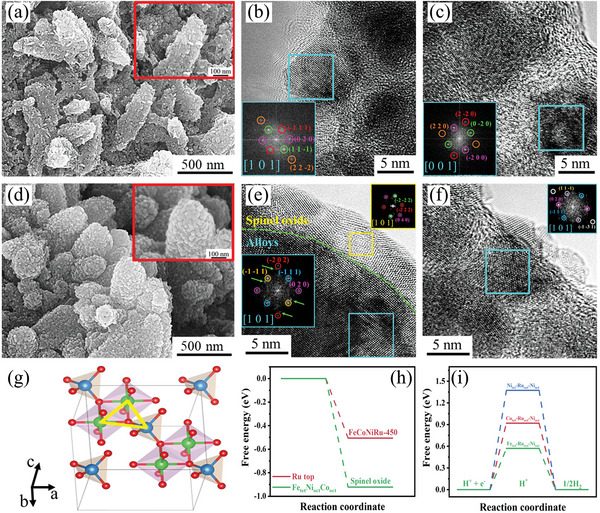
Morphological characterization. a) FESEM and b,c) HRTEM of FeCoNiRu‐450 electrocatalysts after the HER LSV test. d) FESEM and e,f) HRTEM of FeCoNiRu‐450 electrocatalysts after the HER stability test. g) The model of spinel oxide, yellow triangle represent hollow site. h) Water adsorption free energy of Ru top site of FeCoNiRu‐450 and Fe_tet_Ni_oct_Co_oct_ hollow site of spinel oxide, tet, and oct represent tetrahedron and octahedron. i) Hydrogen adsorption free energy of hollow sites with different tetrahedron atoms.

However, the highly efficient catalytic performance of electrocatalysts is not interlinked with the stability in the long‐term run. Catalysis relies on the surface configuration of intrinsic catalysts in contact with electrolytes. This is different from the long‐term stability of catalysts as controlled by the surface self‐reconstruction evolution process when subject to the cyclic potential externally applied. Before the stability test, the morphological and phase structures formed over the surface of the FeCoNiRu‐450 electrocatalyst right after the LSV process were first analyzed by using SEM and transmission electron microscope (TEM) since LSV was reported to enable the activation of the working electrode surface.^[^ ^36^, ^37^, ^38^, ^39^ ^]^ One of the SEM images is shown in Figure S21a, Supporting Information, where the rice‐shaped structures are seen to be uniformly covered across the entire Ni foam surface. These rice‐shaped structures are carbon skeletons as verified from the magnified SEM image (**Figure** **5**a and inset) and are decorated with many nanoparticles. These nanoparticles were further examined in TEM at a high resolution. Figure 5b is one of the TEM images showing the surface lattice structure with different phase contrasts. The fast Fourier transform (FFT) pattern (inset in Figure 5b), a reciprocal image transferred from the nanoparticle marked by the cyan square fully exposed to the electrolyte, is indexed along a zone axis of [1 0 1] and contributed from the pure alloy without being oxidized. The particle remains intact without being destroyed although it is viewed to be very lattice defective as indicated by the IFFT images converted from various lattice planes (Figure S22, Supporting Information). Another particle encapsulated by the layered carbon (Figure 5c) is also remained as the pure alloy demonstrated from the FFT (inset) and is free from oxidization. Likewise, it contains many lattice defects (such as stacking faults and dislocations) as seen from the IFFT images taken from different lattice planes (Figure S23, Supporting Information). These results convinced the fact that the reduction reaction (i.e., HER) occurs on the catalyst surface during LSV.

The HER stability test of FeCoNiRu‐450 was carried out after a longer‐term duration (40 h). In general, the dissolution of electrocatalysts is one of the challenges of holding their stability. Therefore, to clarify that the catalyst is free from dissolution during the stability test, the electrolyte after HER was measured by using ICP‐MS. As shown in Figure S24, Supporting Information, all the elements experience an extremely low dissolution rate after the HER stability test at high current density under the strong alkaline condition, indicating its outstanding compositional stability upon the electrocatalytic process. Such remarkable stability is benefited from the protective carbon skeleton and high corrosion resistance nature of HEAs. Figure S21b, Supporting Information, and Figure 5d are the images taken in SEM at both low and high magnifications. Identical to the LSV test, the rice‐shaped FeCoNiRu‐450 structures are uniformly free‐standing over the surface of Ni foam (Figure S21b, Supporting Information). They maintain their original morphology without collapsing, agglomeration, and peeling off (Figure 5d). This indicates the outstanding structural stability of the FeCoNiRu‐450 electrocatalyst and the high resistance to the strong mechanical impact induced by the bubbling of a large number of hydrogen molecules at high current densities. Meanwhile, the nanoparticles uniformly decorated on the surface of carbon skeletons are still visible and distinct (Figure 5d and inset), which further prove the stability of FeCoNiRu‐450 nanoparticles when suffering from the HER in the alkaline condition. These FeCoNiRu‐450 nanoparticles after the HER stability test were immediately transferred into TEM for additional analysis. Figure 5e is a high‐resolution TEM (HRTEM) where a thin layer (around 5 nm in thickness) is seen to be grown from the original surface of the catalyst. It consists of nanocrystals which are determined to have a spinel structure in the form of AB_2_O_4_ by using the analysis of FFT (yellow square inset in Figure 5e and Figure S26c, Supporting Information) taken along a zone axis of [1 0 1]. This spinel crystal is the result of the long‐term reaction occurring in the interface between the catalyst and alkaline solution. The spinel oxide crystal unit cell is packed by Co, Fe, or Ni atoms that occupy tetrahedron sites, and Ni, Fe, Co, or Ru atoms are positioned at octahedron sites. Ru has the largest atomic size that is incorporated in the spinel lattice and accommodates at the octahedron sites. Co tends to occupy the tetrahedron sites and whilst Ni resides toward octahedron sites. So the compositional phase of the spinel structure in the case of HER is reasonably predicted to be (Fe, Co)(Fe, Ni, Ru)_2_O_4_. This prediction is further proven by the XPS examination. The results show that Fe^2+^ and Fe^3+^ ions remained almost identical across the oxide film (Figure S29a1, Supporting Information). The main valences of Co and Ni are correspondingly associated with +2 and +3 determined from the high‐resolution Co 2p and Ni 2p spectrum (Figure S29b1,c1, Supporting Information). As a consequence, we can claim that the grown thin oxide layer is constructed by the spinel phase (i.e., (Fe, Co)(Fe, Ni, Ru)_2_O_4_). The structure and hollow active sites of spinel oxide are inherited from the original HEA catalyst. In addition, the incorporation of the external elements into the spinel structure will enable the development of the defects and lattice strain over the surface that facilities the HER for high hydrogen production (Figure S26, Supporting Information). It is noted that there are vast carbon‐encapsulated nanoparticles that are not directly in contact with the electrolyte. They have been reported to be equally contributed to HER.^[^ ^40^, ^41^ ^]^ Figure 5f is an example where the particle marked by the cyan square is a carbon‐encapsulated one. It is remained a pure alloy without being oxidized during the long‐term HER process. It is the carbon layer that is responsible for the non‐oxidization of the particle.

To further elucidate the HER performance of the spinel oxide which is in situ formed on the surface of FeCoNiRu‐450 during the HER stability test, DFT calculations were carried out. For spinel oxide, as shown in Figure 5g, the hollow sites consist of two octahedron atoms and one tetrahedron atom. According to the analysis above, Fe, Co, or Ni atoms are positioned at the tetrahedron, and Fe, Co, Ni, or Ru atoms occupy at octahedrons. In the alkaline condition, H_2_O adsorption is the first and critical process of HER. The spinel oxide exhibits a stronger water adsorption ability than FeCoNiRu‐450 due to its larger water adsorption free energy (Tables S12 and S15, Supporting Information, and Figure 5h). More importantly, compared with Ni at the tetrahedron site, almost all the hollow sites show more negative H_2_O adsorption‐free energy when Fe and Co occupy tetrahedron (Table S15, Supporting Information), verifying that the hollow sites have stronger H_2_O adsorption ability when Fe and Co are located at tetrahedron. This is also in agreement with the experiment results that Fe and Co tend to occupy tetrahedron sites more preferentially than Ni during the surface self‐reconstruction process. The same results are also discovered for hydrogen adsorption‐free energy. For all the hollow sites, compared with Ni positioning at tetrahedron sites, the occupation of Fe and Co at tetrahedron sites brings about the hydrogen adsorption‐free energy closer to zero (Table S16, Supporting Information, and Figure 5i). It consequently makes more efficient hydrogen adsorption and desorption process during HER, leading to extremely high electrocatalytic HER activity of the electrocatalyst.^[^ ^42^, ^43^ ^]^ These results demonstrate that the long‐term HER stability of FeCoNiRu‐450 originates from the surface self‐reconstruction process.

### 2.7 Surface Structures of the FeCoNiRu‐450 Electrocatalyst upon the Long‐Term Oxygen Evolution Reaction Stability Test

**Figure 6:**
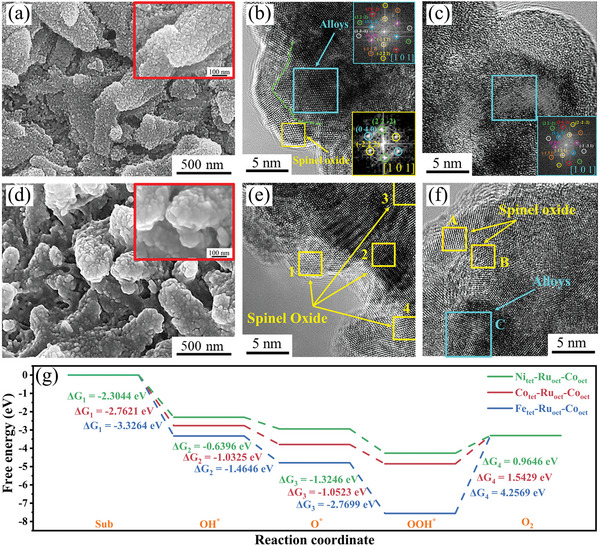
Morphological characterization. a) FESEM and b,c) HRTEM of FeCoNiRu‐450 electrocatalysts after the OER LSV test. d) FESEM and e,f) HRTEM of FeCoNiRu‐450 electrocatalysts after the OER stability test. g) OER energy diagram of hollow sites with different tetrahedron atoms.

Based on the experiments above, the FeCoNiRu‐450 electrocatalyst can provide equivalently excellent OER stability. To activate the FeCoNiRu‐450 surface, the LSV test for OER was also carried out first before the stability assessment, which is subsequently observed in SEM and TEM. Figure S31a, Supporting Information, and **Figure** **6**a display two images with one taken at low magnification and the other at high magnification respectively. Figure S31a, Supporting Information, presents a uniform dispersion of rice‐shaped structures which are again carbon skeletons. These skeletons appear in their original geometry without any deterioration. Their surface are seen with the decoration of nanoparticles (Figure 6a and inset). These results indicate the initial stability of the FeCoNiRu‐450 electrocatalyst in response to the OER LSV test. The two types of nanoparticles are also visualized through the surface self‐reconstruction process of FeCoNiRu‐450 nanoparticles under the OER test. The nanoparticle is the one exposed to the electrolyte which is coated by a thin layer (Figure 6b). The growth of the thin oxide layer is resulted from the surface self‐reconstruction process of the FeCoNiRu‐450 when it undergoes the OER process. The oxide is identified to be also a spinel structure by using HRTEM through the analysis of the FFT pattern (yellow square inset in Figure 6b). Figure 6c shows the other type of nanoparticle which is marked by the cyan square. The inset is the corresponding FFT pattern, which is generated from the pure alloy without being oxidized. It is the carbon layer that prevents the alloy from oxidization.

When FeCoNiRu‐450 experiences a long‐term OER stability test, the electrolyte was measured using ICP‐MS to determine the dissolution rate of all the elements (Figure S24, Supporting Information). The results show the extremely low dissolution rate of all the elements after the OER long‐term stability test at high current density under the strong alkaline condition, indicating its brilliant compositional stability of the electrocatalytic process. This excellent stability should be beneficial from the inert carbon skeleton and HEA itself with high corrosion resistance. After the OER long‐term stability test, the surface of FeCoNiRu‐450 turns to Figure 6d–f and shows the morphology (i.e., the carbon skeleton distribution and geometrical shape) that is almost identical to the previous surface after the LSV test (Figure S31b, Supporting Information, and Figure 6d). Nanoparticles are also uniformly dispersed across the carbon skeletons (Figure 6d and inset) and fully oxidized. The fairly smooth surface in Figure 6d and the inset is the evidence that a thin oxide layer seems to cover the entire surface of the carbon skeleton. This oxide layer is further determined from Figure 6e where all catalyst nanoparticles have been oxidized (such as those yellow square marked areas: 1–4). Same as the LSV test, the oxide is a spinel structure, determined from the FFTs of 1’‐ 4,’ A,’ and B’ (the corresponding FFTs pattern shown in Figures S35–S40, Supporting Information). It consists of spinel nanocrystals which have a structure in the form of AB_2_O_4_ as proven by the FFT (Figures S35–S40, Supporting Information). The spinel oxide crystal unit cell is packed by Co, Fe, or Ni atoms that occupy tetrahedron sites and Ni, Fe, Co, or Ru atoms positioned at octahedron sites. Ru has the largest atomic size that is incorporated in the spinel lattice and accommodates at the octahedron sites. Co and Ni tend to occupy the opposite sites after HER, that is, the octahedron and tetrahedron sites, respectively. As a result, the compositional phase of the spinel structure in the case of OER is reasonably identified to be (Fe, Ni)(Fe, Co, Ru)_2_O_4_, as further verified by the XPS analysis. The results show that Fe^2+^ and Fe^3+^ ions are almost identical across the oxide film (Figure S43a1, Supporting Information). The main valences of Co and Ni are correspondingly associated with +3 and +2 determined from the high‐resolution Co 2p and Ni 2p spectrum (Figure S43b1,c1, Supporting Information). The nanoparticle “C” in Figure 6f remains the pure alloy without oxidization as verified from the FFT pattern (Figure S41, Supporting Information). As such, it is reasonable to estimate the distance between the edge and the left surface of particle C as the oxide surface, which is measured to be ≈10 nm.

DFT calculations were used to further clarify the OER performance of the spinel oxide which is in situ formed on the surface of FeCoNiRu‐450 during the OER stability test. For OER in alkaline conditions, the lower OH^−^ adsorption free energy corresponds to the smaller O_2_ desorption free energy, demonstrating better electrocatalytic OER performance. Therefore, the OH^−^ adsorption free energy of all the hollow sites was calculated. Compared with Fe and Co occupying tetrahedron sites, almost all the hollow sites show lower OH^−^ adsorption free energy when Ni is positioned at tetrahedron sites (Table S17, Supporting Information). It is also in agreement with the experiment results that Ni tends to occupy tetrahedron sites more preferentially than Fe and Co during the surface self‐reconstruction process. In addition, due to both Co and Ru that tend to position at octahedron, the free energy of the entire OER reaction path for Ni_tet_‐Ru_oct_‐Co_oct_, Co_tet_‐Ru_oct_‐Co_oct_, and Fe_tet_‐Ru_oct_‐Co_oct_ hollow sites were calculated. As shown in Figure 6g, Ni_tet_‐Ru_oct_‐Co_oct_ hollow site presents the lowest OH^−^ adsorption free energy (Δ*G*_1_) and O_2_ desorption free energy (Δ*G*_4_) over others, indicating that this hollow site exhibits the best electrocatalytic OER performance. It is also consistent with the analysis above. Moreover, Ni_tet_‐Ru_oct_‐Co_oct_ hollow site was calculated to have the smallest free energy change at the rate‐determining step, further suggesting that this hollow site with Ni occupying the tetrahedral sites tops the OER of the HEA (Figure 6g). Furthermore, except for the slightly higher OOH* reaction formation free energy than Co_tet_‐Ru_oct_‐Co_oct_ hollow site, Ni_tet_‐Ru_oct_‐Co_oct_ hollow site presents the lowest reaction intermediates (OH*, O*, and OOH*) formation free energy over other hollow sites (Figure 6g). This fact demonstrates that Ni occupying tetrahedron sites can accelerate the entire OER reaction pathway. These DFT calculations unravel that the surface self‐reconstruction process enables the long‐term sustainable OER stability of FeCoNiRu‐450.

## 3 Conclusion 

In summary, FeCoNiRu HEA electrocatalyst is successfully prepared and evaluated to provide both high catalytic efficiency and excellent long‐term stability for water redox. The results reveal that the intrinsic hollow active sites dispersed over the surface of the catalyst contributed to the high performances of HER and OER. However, the same hollow active sites preserved in the spinel structure are inherited from the original HEA catalyst when subject to the external potential/current applied. They are susceptible to H^+^ and OH^−^ and facilitate the corresponding HER and OER. This unravels that the catalyst constantly remains high catalytic activity after 40 h's stability test. In addition, the accommodation of Ru in the catalyst brings about the distortion of the surface lattice as well as defects that also speeds up the HER and OER. It is believed that this work will provide new insight into the surface self‐reconstruction of electrocatalysts under HER and OER reaction conditions and a benchmark for the evaluation of the efficiency–stability relationship.

## 4 Experimental Section

### Experimental Procedures

Chemicals and Reagents: Iron acetate tetrahydrate (Fe(CO_2_CH_3_)_2_·4H_2_O), cobalt nitrate hexahydrate (Co(NO_3_)_2_·6H_2_O), ruthenium acetylacetonate (C_15_H_21_O_6_Ru), *N*,*N*‐Dimethylformamide (DMF, C_3_H_7_NO), potassium hydroxide (KOH), the commercial Pt/C 20 wt% catalyst, and the commercial RuO_2_ catalyst were purchased from Shanghai Macklin Biochemical Co. Nickel nitrate hexahydrate (Ni(NO_3_)_2_·6H_2_O), isopropanol (C_3_H_8_O), and anhydrous ethanol (C_2_H_6_O) were bought from Beijing Tongguang Fine Chemicals Company. 5 wt% Nafion solution, and 2,5‐dihydroxyterephthalic acid (C_8_H_6_O_6_) were obtained from Sigma‐Aldrich. Hydrochloric acid was acquired from Sinopharm chemical reagent Co. Ltd. All the reagents were used as received without any further purification.

### Synthesis of Quaternary FeCoNiRu High‐Entropy Metal‐Organic Frameworks Precursor

Before the synthesis, the purchased Ni foam (NF) was washed in dilute hydrochloric acid to remove the oxide layer on their surface. The NF was then ultrasonic cleaned in deionized water and transferred in anhydrous ethanol to repeat ultrasonic cleaning, followed by drying. The synthesis of quaternary HEMOFs/NF started with the dissolution of 0.06 mmol of Fe(CO_2_CH_3_)_2_·4H_2_O, 0.06 mmol of Co(NO_3_)_2_·6H_2_O, 0.06 mmol of Ni(NO_3_)_2_·6H_2_O, 0.10 mmol of C_15_H_21_O_6_Ru, and 0.10 mmol of C_8_H_6_O_6_ mixed with 4.5 mL of DMF, 0.27 mL of anhydrous ethanol, and 0.27 mL of deionized water solvent for at least 30 min with sonication forming a uniform solution. Subsequently, the solution was immediately transferred into a 10 mL autoclave with a piece of acid‐treated NF fixed, which was followed by vertically immersing it into the solution. After that, the autoclave was heated to 150 °C and remained for 48 h allowing the solvothermal reaction. Finally, the quaternary HEMOFs/NF products were washed with DI water, DMF, and EtOH in sequence several times and dried at 60 °C.

Synthesis of unary, binary, and ternary MOFs precursor was the same as the synthesis of quaternary HEMOFs, addition of corresponding precursor salt, respectively.

### Synthesis of FeCoNiRu High‐Entropy Alloys

Synthesis of FeCoNiRu HEAs nanoparticles@N‐doped porous carbon/NF: FeCoNiRu‐X (X = 1, 2, 3, 4, 5) were carried out through the pyrolysis of HEMOFs/NF precursors in a tube furnace at different temperatures. The HEMOFs/NF precursors were first pretreated at 350 °C for 1 h, and then went on heating at different temperatures (i.e., 350, 400, 450, 500, and 550 °C, denoted as FeCoNiRu‐350, FeCoNiRu‐400, FeCoNiRu‐450, FeCoNiRu‐500, and FeCoNiRu‐550, respectively) for carbonization and maintained at each temperature for 2 h with a ramp rate of 5 °C min^−1^ and under a mixed gas H_2_/Ar (5% H_2_) flow. Note that the loading mass of active material of FeCoNiRu‐350, FeCoNiRu‐400, FeCoNiRu‐450, and FeCoNiRu‐500 composites on NF was estimated to be ≈2.00, ≈0.65, ≈0.34, and ≈0.19 mg cm^−2^, respectively.

Synthesis of unary, binary, and ternary alloys followed the same procedure steps as the synthesis of FeCoNiRu‐450 apart from the alternation of different precursor templates in a tube furnace.

### Material Characterization

The morphologies of the synthesized materials were examined using field‐emission scanning electron microscopy (Regulus 8100, Hitachi, Japan). The HAADF‐STEM and energy‐dispersive X‐ray spectroscopy mapping were performed by a JEOL JEM‐ARM 200F with double spherical aberration correctors. XRD patterns were collected using an X‐ray diffractometer (Ultima IV, Rigaku, Japan) with Cu K*α* radiation (*λ* = 1.5406 Å). Raman measurements were performed on a Raman spectrometer (HR800, Horiba Jobin‐Yvon, France) using a 532 nm laser. The specific surface area was determined from the results of N_2_ physisorption at 77 K (Micromeritics ASAP 2460, Micromeritics instrument corporation, USA) by using the BET (Brunauer‐Emmet‐Teller). XPS was recorded using an X‐ray photoelectron spectrometer (Escalab Xi+, Thermo Fisher Scientific, USA), with non‐monochromatized Al K*α* X‐rays as the excitation source. The content of metal elements was measured using an ICP‐MS (icap 6300, Thermo Fisher Scientific, USA).

### Electrochemical Measurements

All the electrochemical measurements were conducted on an electrochemical workstation (Autolab PGSTAT 302N) with a standard three‐electrode setup at room temperature where the as‐prepared electrocatalyst materials were used as the working electrode and the graphite rod and Ag/AgCl electrode in saturated KCl served as the counter and reference electrodes, respectively. It should be noticed, to prepare the commercial Pt/C and RuO_2_ electrocatalyst as the working electrode for comparison, 5 mg catalysts were dispersed in a mixed solvent that contains 360 µL deionized water, 120 µL isopropanol, and 20 µL Nafion (5%), followed by sonication for at least 30 min forming a homogeneous ink. The catalyst ink was then loaded onto the surface of NF followed by drying at room temperature.

The HER and OER performances were evaluated in N_2_‐saturated and O_2_‐saturated 1 m KOH solution, respectively. The electrocatalytic activity of the as‐prepared samples was examined by LSV at a scan rate of 10 mV s^−1^. All the measured LSV potentials in this work were performed with iR compensation (90%). The EIS spectra were collected in a frequency range of 100 kHz to 0.01 Hz at an overpotential corresponding to 10 mA cm^−2^ current density. The cycle durability was measured by cyclic voltammetry (CV, sweep rate, 100 mV s^−1^) and a chronoamperometric response.

### Overall Water Splitting

The overall water splitting was recorded in a two‐electrode system in 1 m KOH solution. The FeCoNiRu‐450 served as both negative and positive electrodes for HER and OER. For comparison, the commercial Pt/C and RuO_2_ electrocatalyst load on the surface of NF as negative and positive electrodes for HER and OER, respectively. The commercial Pt/C and RuO_2_ electrocatalyst loadings are the same (i.e., 0.34 mg cm^−2^).

The electrochemical active surface area (ECSA) of the catalyst was estimated using the following equation:

(1) $$\mathrm{ECSA}=C_{\mathrm{dl}}C_{s}$$

where *C*_dl_ and *C*_s_ represent the double‐layer capacitance and specific capacitance, respectively. The *C*_dl_ value was measured from cyclic voltammetry (CV) curves at different scan rates (10, 20, 30, 40, 50 mV s^−1^) within the non‐faradaic potential range of 0.05–0.10 V versus RHE, and by plotting Δ*j* = (*j*_a_ − *j*_c_)/2 at 0.075 V versus RHE as a function of the scan rate. The slope of the linear fit corresponds to the *C*_dl_ value.

The TOF values were calculated from the equation:

(2) $$\mathrm{TOF}=\frac{JA}{2Fn}\left(\mathrm{HER}\right)$$

(3) $$\mathrm{TOF}=\frac{JA}{4Fn}\left(\mathrm{OER}\right)$$

where *J* is the current density at a certain overpotential, *A* is the surface area of the electrode, 2 and 4 is the mole of electrons transferred to generate one mole of H_2_ and O_2_, respectively, *F* is the Faraday constant (96 485 C mol^−1^), and *n* is the number of moles of active sites. TOFs were recorded based on the assumption that all transition metal atoms in the samples are catalytically active.

### Density Functional Theory Calculations

The DFT computations were obtained with the GGA‐PBE exchange‐correlation functional^[^ ^44^ ^]^ as implemented in the Vienna Ab initio simulation package.^[^ ^45^ ^]^ The initial slab model of the FCC HEA containing 64 atoms (Ru:Ni:Co:Fe = 7:21:21:15) was constructed by randomly substituting elements. A hybrid Monte‐Carlo molecular‐dynamics was then performed to search the stable distributions of elements. Monte Carlo swaps of atoms were performed at each of 10 molecular‐dynamics steps and the swap probabilities were dictated by a Metropolis criterion. The slab model obtained from the final structure after 5000 steps is shown in Figure S20, Supporting Information. The interaction between valence electrons and ionic cores was described by projected augmented waves with a cut‐off energy of 400 eV. 3 × 3 × 1 Gamma‐centered Monkhorst‐Pack k‐grids are employed during calculations. The convergence threshold was set as 1.0 e^−6^ eV in and 0.02 eV Å^−1^ in force, respectively.

In addition, unary Co, binary FeCo, and ternary FeCoNi alloys were also calculated using the same method with FeCoNiRu HEA. The slab model contains 64 atoms with the atoms ratio of Fe:Co = 1:1 for binary FeCo alloy and Fe:Co:Ni = 1:1:1 for ternary FeCoNi alloy.

### The Calculation of Free Energies

The adsorption‐free energy of H/H_2_O (Δ*G*_M*_, M represent H or H_2_O) is usually used to evaluate the HER activity, and is calculated by

(4) $$\Delta G_{M^{*}}=\Delta E_{M^{*}}+\Delta \mathrm{ZPE}-T\Delta S$$

where the respective Δ*E*_M*_, ΔZPE, and Δ*S* represent the binding energy, zero point energy change, and entropy change of H* or H_2_O* adsorption. The $\Delta G_{H_{2}O*}$ at each top site and Δ*G*_H*_ at each hollow site are scanned as shown in Figure 4a,b and Tables S12 and S13, Supporting Information.

The adsorption‐free energy of OH (Δ*G*_OH*_) is calculated by

(5) $$\Delta G_{\mathrm{OH}*}=\Delta E_{\mathrm{OH}*}-\mu_{H_{2}O}+\frac{1}{2}\mu_{H_{2}}+\Delta G$$

where Δ*E*_OH*_ and Δ*G* represent the respective binding energy of OH and relative contributions from vibrational energy including zero point energy and internal energy. $\mu_{H_{2}O}$ and $\mu_{H_{2}}$ represent the corresponding free energies of H_2_O and H_2_ molecules. The Δ*G*_OH*_ at Ru top sites and each hollow site is scanned as shown in Figure 4f and Table S14, Supporting Information. The whole standard free energy diagram for the OER with *U* = 1.23 eV and pH = 14 is calculated according to the refs. [46, 47].

## Conflict of Interest

The authors declare no conflict of interest.

## Author Contributions

K.H., J.X., and Y.L. contributed equally to this work. K.H., B.Z., J.W., and Y.H. designed the study. K.H. and J.X. performed the experiments and analyzed the data. K.H. and Y.L. drafted the manuscript. W.S. and C.C. set up the model and execute the DFT calculations. X.C., X.Z., L.M.W., and T.W. implemented the analysis of experimental and DFT calculation data. J.W. and Y.H. supervised the research. All authors discussed the results and revised the manuscript.

## Supporting information

Supporting Information

## Data Availability

The data that support the findings of this study are available from the corresponding author upon reasonable request.
