# Supplementary material for: Self‐Reconstructed Spinel Surface Structure Enabling the Long‐Term Stable Hydrogen Evolution Reaction/Oxygen Evolution Reaction Efficiency of FeCoNiRu High‐Entropy Alloyed Electrocatalyst
Source: Adv Sci (Weinh). 2023 Mar 22;10(14):2300094. doi: 10.1002/advs.202300094 (PMC10190517; doi:10.1002/advs.202300094)
Supplement: Supplementary file 1 — Supporting Information [file ADVS-10-2300094-s001.pdf]

ADVANCED SCIENCE
Open Access

Supporting Information

for *Adv. Sci.*, DOI 10.1002/advs.202300094

Self-Reconstructed Spinel Surface Structure Enabling the Long-Term Stable Hydrogen Evolution Reaction/Oxygen Evolution Reaction Efficiency of FeCoNiRu High-Entropy Alloyed Electrocatalyst

Kang Huang, Jiuyang Xia, Yu Lu, Bowei Zhang, Wencong Shi, Xun Cao, Xinyue Zhang, Lilia M. Woods, Changcun Han, Chunjin Chen*, Tian Wang, Junsheng Wu* and Yizhong Huang**

Self-reconstructed spinel surface structure enabling the long-term stable HER/OER efficiency of FeCoNiRu high-entropy alloyed electrocatalyst

Kang Huang, Jiuyang Xia, Yu Lu, Bowei Zhang*, Wencong Shi, Xun Cao, Xinyue Zhang, Lilia M. Woods, Changcun Han, Chunjin Chen*, Tian Wang, Junsheng Wu*, Yizhong Huang*

K. Huang, J. Xia, B. Zhang, J. Wu
Institute for Advanced Materials and Technology
University of Science and Technology Beijing
Beijing, 100083, China
E-mail: bwzhang@ustb.edu.cn; wujs76@163.com

Y. Lu, X. Cao, X. Zhang, Y. Huang
School of Materials Science and Engineering
Nanyang Technological University
50 Nanyang Avenue, 639798, Singapore
E-mail: yzhuang@ntu.edu.sg

W. Shi
School of Biological Sciences
Nanyang Technological University
50 Nanyang Avenue,639798, Singapore

L. Woods
Department of Physics
University of South Florida
Tampa, Florida 33620, USA

C. Han, Y. Huang
College of Science
Hubei University of Technology
Wuhan, 430068, China

C. Chen
Institute of Metal Research
Chinese Academy of Sciences
Shenyang, 110016, China
E-mail: cjchen16s@imr.ac.cn

T. Wang
Department of Chemistry
National University of Singapore
3 Science Drive 3, 117543, Singapore

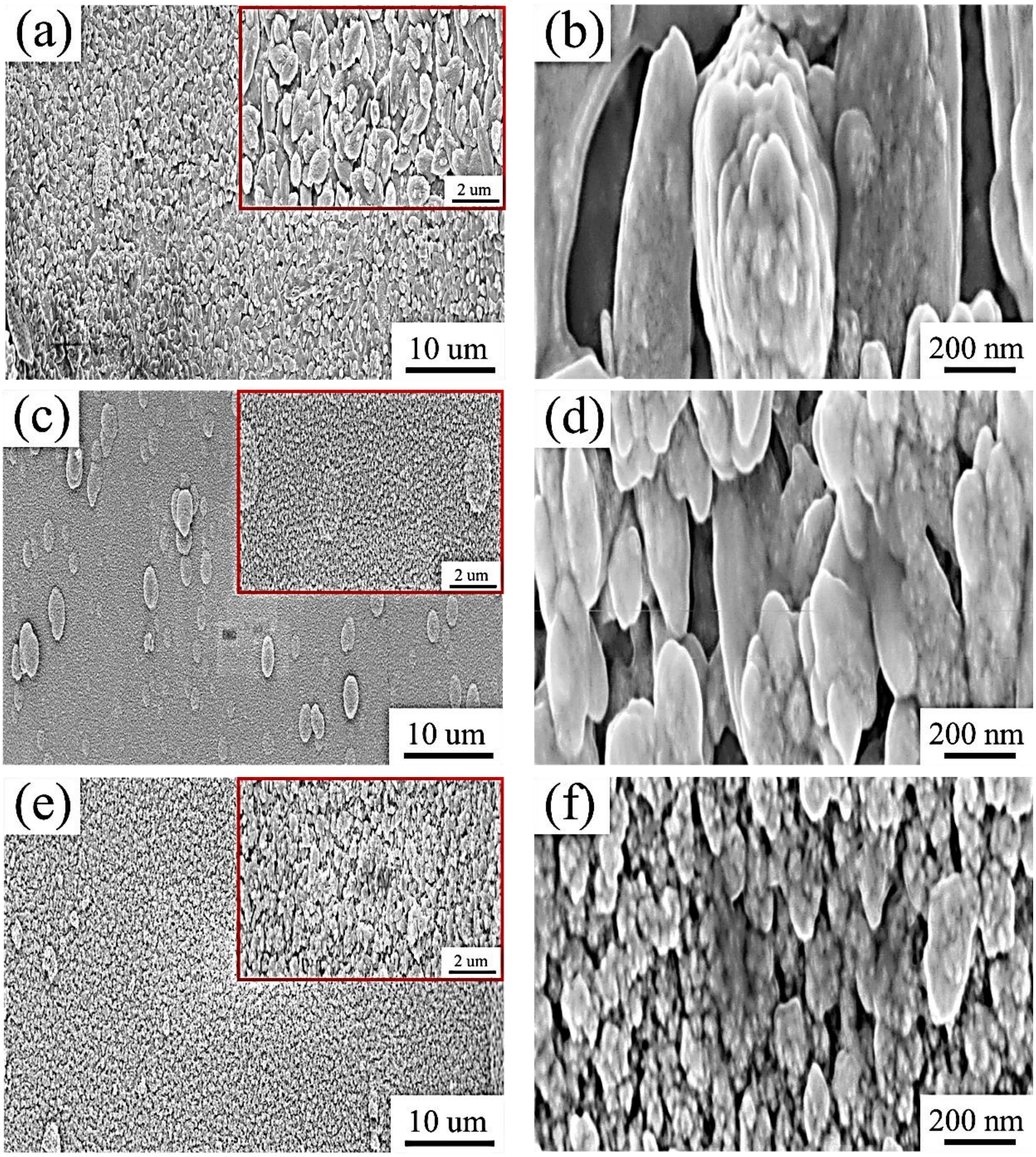

Figure S1 Morphological characterization. Low and high magnification FESEM images of HEMOFs precursor synthesized at different solvothermal reaction times. (a, b) 6 h; (c, d) 12 h; and (e, f) 24 h.

Figure S1 and Figure1b and c show the changeable morphologies of quaternary FeCoNiRu HEMOFs precursors synthesized with different reaction times. Thus, the reaction time is a critical parameter that influences the morphology of the HEMOFs precursor.

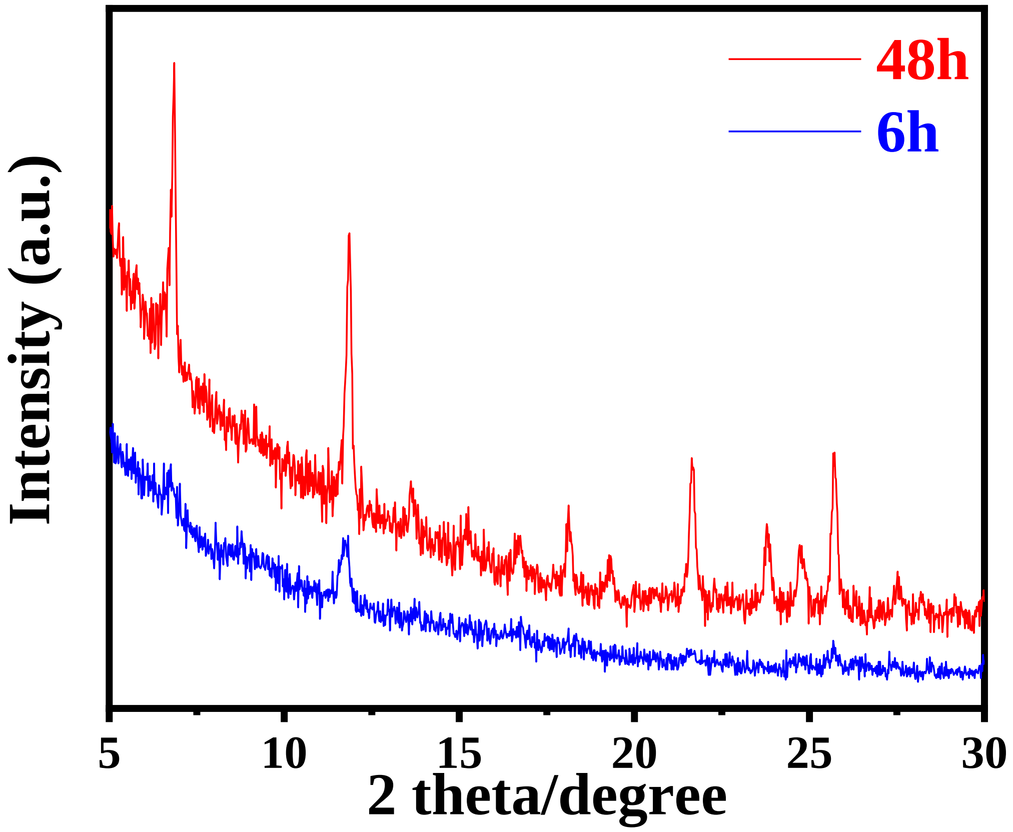

Figure S2 Phase structure characterization. X-ray diffraction (XRD) patterns of HEMOFs precursor synthesized at different solvothermal reaction times.

Figure S2 shows the different XRD patterns of HEMOFs precursor synthesized at different reaction times. A better crystallinity is obtained after 48 h reaction compared to the 6 h. Thus, the reaction time is a parameter that also needs to be considered during the synthesis of HEMOFs precursor.

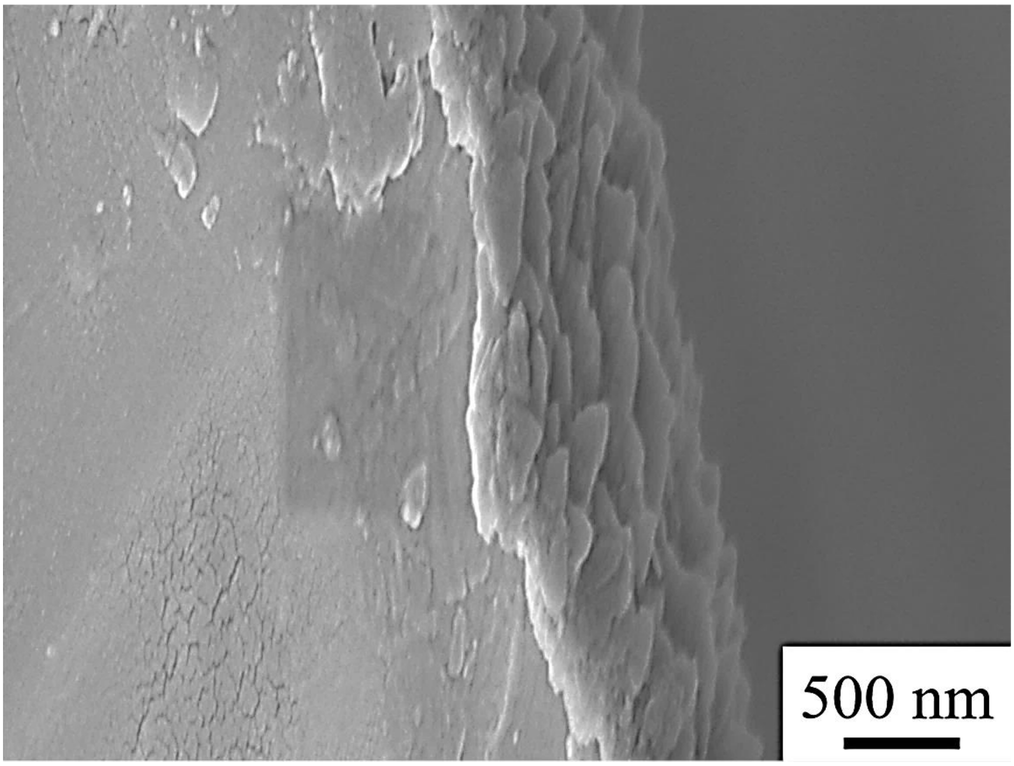

Figure S3 Morphological characterization. A cross-sectional image of HEMOFs precursor.

The cross-sectional image in Figure S3 illustrates that HEMOFs precursors are uniformly attached to the Ni substrate proving that the HEMOFs have extremely high purity without any other impurities.

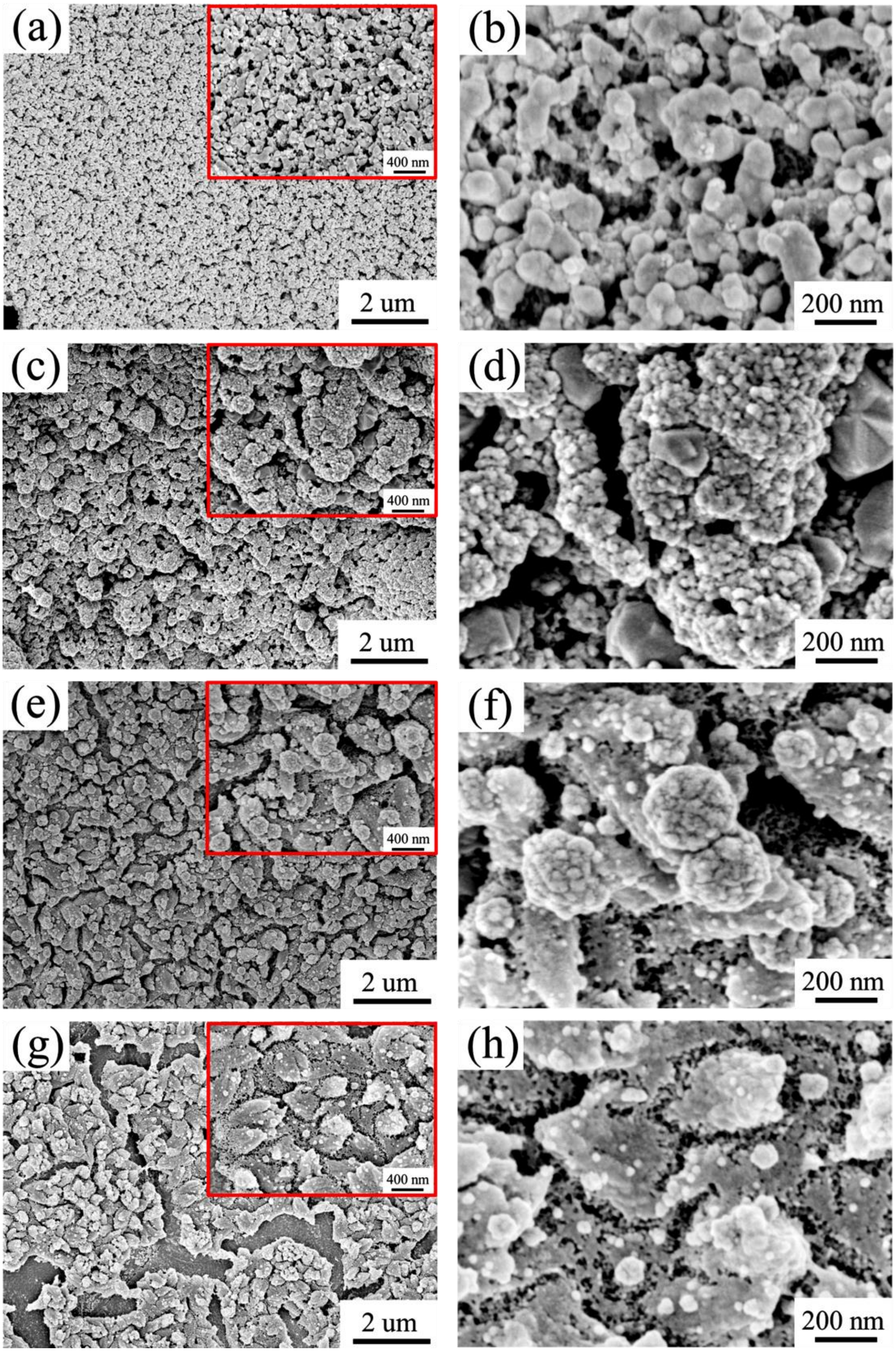

Figure S4 Morphological characterization of FeCoNiRu-X (X = 350, 400, 500, and 550). Low and high magnification FESEM images of (a, b) FeCoNiRu-350; (c, d) FeCoNiRu-400; (e, f) FeCoNiRu-500; and (g, h) FeCoNiRu-550.

Figure S4 and Figure 1e and f show the morphologies of FeCoNiRu HEMOFs precursor after pyrolysis at different parameters. Figure S4a and b show that FeCoNiRu HEMOFs are almost not decomposed after pyrolysis at 350 °C for 3 h (denote: FeCoNiRu-350). Figure S4c and d show that the HEMOFs precursor is not completely decomposed after pyrolysis at 350 °C for 1 h followed by 400 °C for 2 h (denote: FeCoNiRu-400). However, Figure S4e-h presents the collapse and agglomeration of FeCoNiRu HEMOFs (denote: FeCoNiRu-500 and FeCoNiRu-550) when the temperature increases to 500 and 550 °C. For comparison, Figure 1e and f show that FeCoNiRu HEMOFs are fully decomposed without collapse and agglomeration after pyrolysis at 350 °C for 1 h and then 450 °C for 2 h (denote: FeCoNiRu-450).

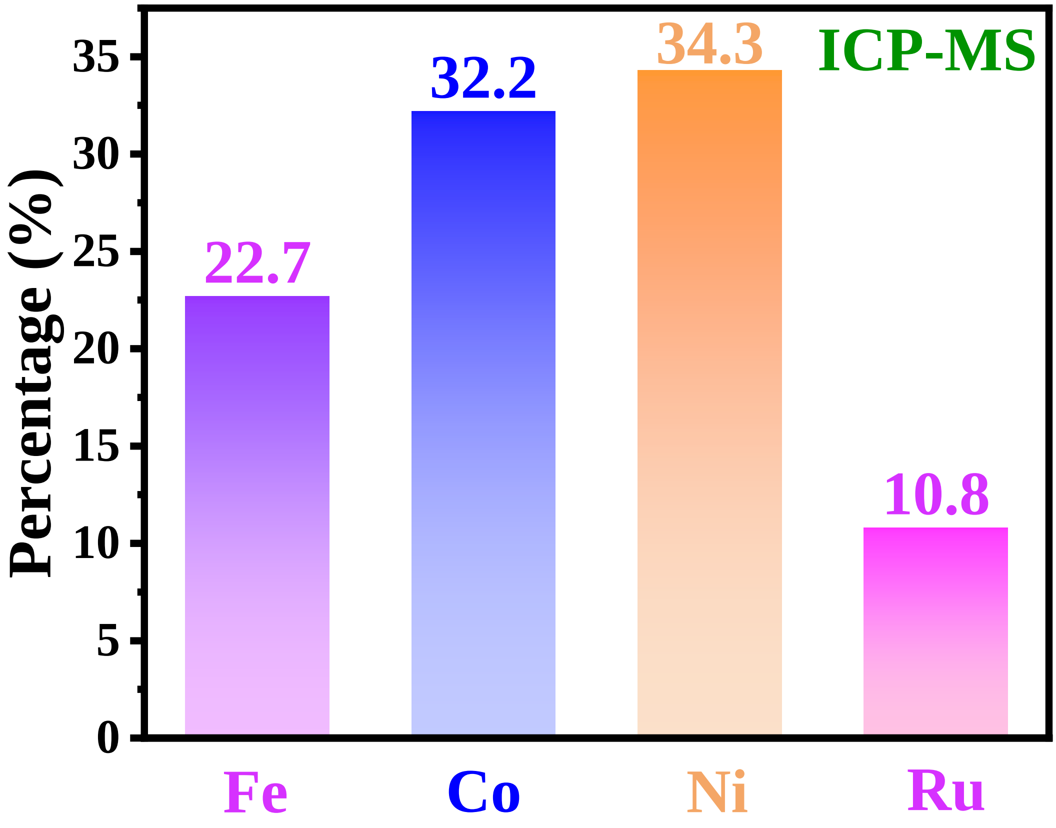

Figure S5 ICP-MS of FeCoNiRu-450 nanoparticles and corresponding element contents of the FeCoNiRu-450 catalyst.

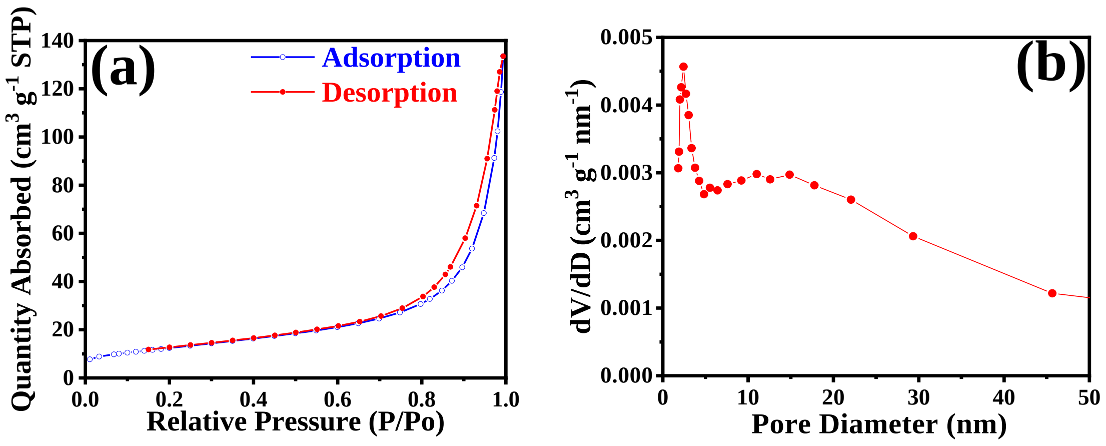

Figure S6 BET and porosity analysis of FeCoNiRu-450. (a) N_2 ad-/desorption isotherms, (b) Pore size distribution of FeCoNiRu-450.

Figure S6 shows the typical type IV isotherm with a distinct hysteresis loop indicating that the FeCoNiRu-450 possesses a large Brunauer-Emmett-Teller (BET) surface area of 44.8 m^2 g^{-1} with an abundant mesoporous structure. The pore size distribution curves suggest the coexistence of micropores and mesopores in FeCoNiRu-450. The high BET surface area is expected to supply more catalytic active sites and contact areas, and the porosity is beneficial for charge and mass transport during electrocatalysis.

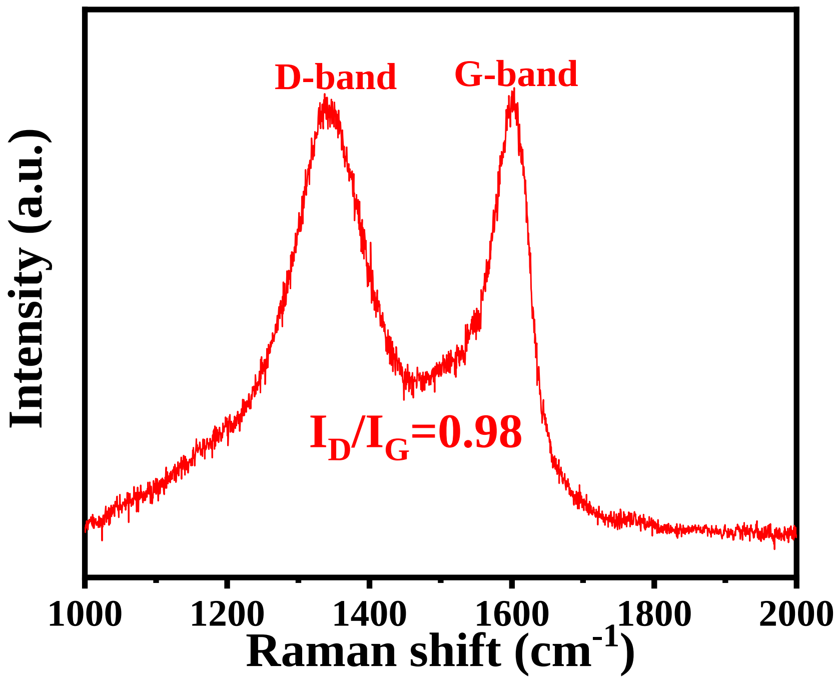

Figure S7 Carbon structure analysis of FeCoNiRu-450. Raman spectrum of FeCoNiRu-450.

The carbon structure of FeCoNiRu-450 was analyzed using a Raman spectrum. Two peaks located at 1340 cm^{-1} and 1603 cm^{-1} are assigned to typical D and G bands of carbon, which correspond to the defects and graphitization degree of carbon materials, respectively. The high intensity of the G band represents a high graphitization degree, which is favorable to the electronic conductivity. The high I_D/I_G value indicates more defect numbers in the carbon frameworks of FeCoNiRu-450, offering more catalytic reaction active sites. In addition, the upshift of the G band may be due to the formation of more nanocrystalline graphite in carbon frameworks.[1]

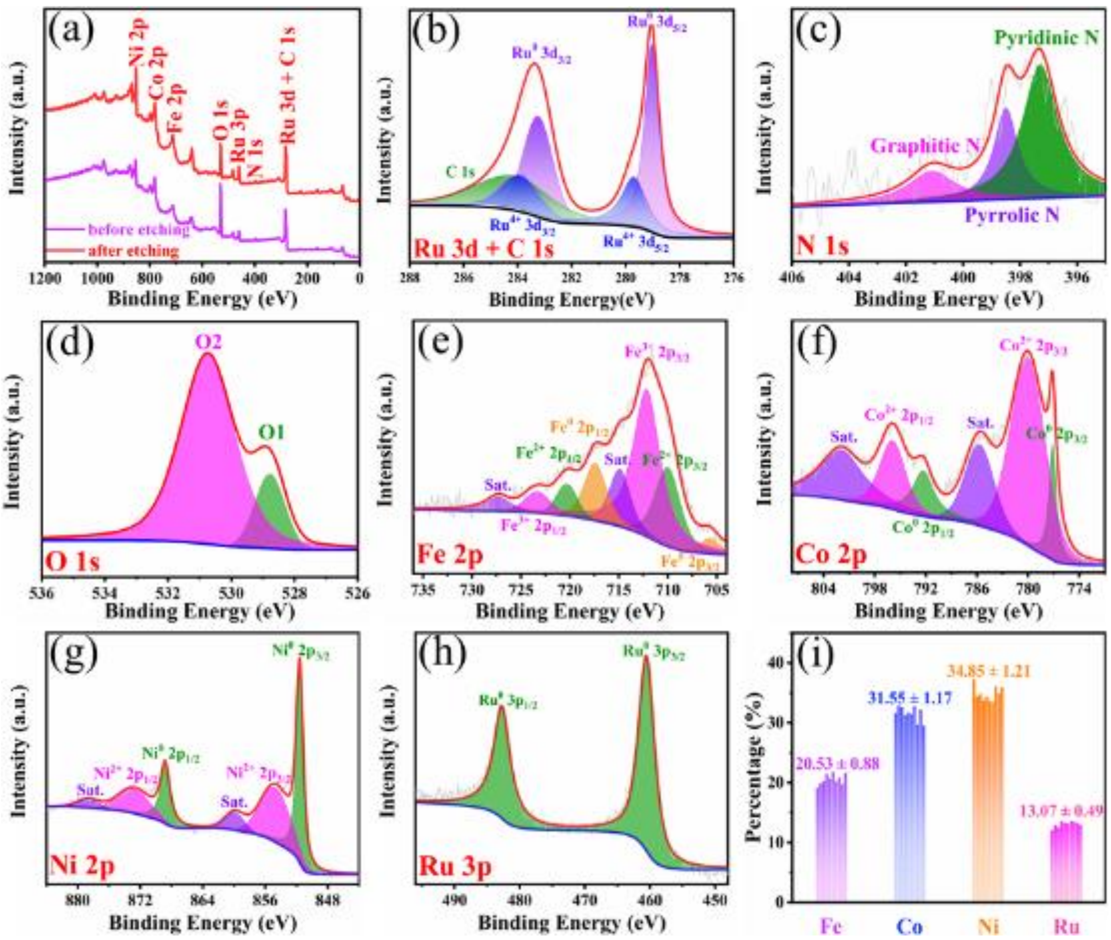

Figure S8 XPS compositional analysis of FeCoNiRu-450 nanoparticles. (a) XPS spectra of FeCoNiRu-450 nanoparticles before and after etching, (b-h) Ru 3d + C 1s, N 1s, O 1s, Fe 2p, Co 2p, Ni 2p, and Ru 3p spectra, and (i) each element atom percentage.

The chemical and electronic states of FeCoNiRu-450 were investigated by X-ray photoelectron spectroscopy (XPS). The results suggest that the FeCoNiRu-450 is oxidized in the air due to the oxygen affiliation nature of transition metals. Therefore, the etching process of XPS was carried out to acquire the real surface chemical and electronic state information of the FeCoNiRu-450. The XPS survey spectra of the FeCoNiRu-450 before and after etching present a series of peaks corresponding to Ru 3d, C 1s, N 1s, Ru 3p, O 1s, Fe 2p, Co 2p, and Ni 2p (Figure S8a). The intensity of peaks of O 1s is noted to reduce after the etching process, suggesting the existence of oxygen on the surface of FeCoNiRu-450. Meanwhile, after the etching process, all the maximum peak intensities of Fe 2p, Co 2p, and Ni 2p shift to lower binding energy, indicating that the etching process undermines the valent state of all transition metal

elements. This can also prove that oxidation only happens on the surface of FeCoNiRu-450. The high-resolution N 1s spectrum can be deconvoluted into three peaks at 397.3 eV, 398.5 eV, and 401.1 eV (Figure S8c), which are attributed to pyridinic N, pyrrolic N, and graphite N, respectively. The pyrrolic N and pyridinic N species can improve the electrocatalytic activity by interacting with H^+ for the HER.[2] The graphite N can increase electrical conductivity.[3] Therefore, the N atoms doping into carbon frameworks increase additional defect catalytic active sites and promote the electrical conductivity, leading to the enhancement of the electrocatalytic performance. The high-resolution O 1s spectrum can be fitted into two peaks at 528.9 eV and 530.7 eV (Figure S8d), which are attributed to the lattice oxygen (O1) and surface adsorbed oxygen-containing species (O2), respectively. The high-resolution Fe 2p spectrum signifies the presence of metallic Fe and iron oxide (705.7 eV for Fe^0 $2p_{3/2}$, 710.0 eV for Fe^{2+} $2p_{3/2}$, 712.2 eV for Fe^{3+} $2p_{3/2}$, 717.4 eV for Fe^0 $2p_{1/2}$, 720.3 eV for Fe^{2+} $2p_{1/2}$, and 723.3 eV for Fe^{3+} $2p_{1/2}$, Figure S8e). The high-resolution Co 2p spectrum also includes a zero-valence state (777.1 eV for Co^0 $2p_{3/2}$ and 792.3 eV for Co^0 $2p_{1/2}$), and the peaks centered at 780.0 eV and 795.9 eV are attributed to Co^{2+} $2p_{3/2}$ and Co^{2+} $2p_{1/2}$ (Figure S8f). The high-resolution Ni 2p spectrum shows the high peak intensity of the zero-valence state at 851.6 eV (Ni^0 $2p_{3/2}$) and 868.8 eV (Ni^0 $2p_{1/2}$), and the peaks at 854.7 eV and 872.8 eV correspond to Ni^{2+} $2p_{3/2}$ and Ni^{2+} $2p_{1/2}$ (Figure S8g). The high-resolution Ru 3p spectrum can be only fitted into two peaks at 460.5 eV and 482.7 eV, which are generated from Ru $3p_{3/2}$ and Ru $3p_{1/2}$ (Figure S8h). The combined analysis of high-resolution spectra for Fe, Co, Ni, and Ru demonstrates that the oxidization degree of each element in FeCoNiRu-450 is in connection with its chemical activity. In addition, the content of each element of FeCoNiRu-450 calculated by XPS is shown in Figure S8i, which is consistent with the ICP-MS results shown in Figure S5.

Table S1. Comparison of HER performance of FeCoNiRu-450 electrocatalyst with recently reported HEAs and other noble metal-based HER electrocatalysts.

**Materials**	**Electrolyte (KOH)**	**η_{10} (mV)**	**Tafel slope (mV dec^{-1})**	**Ref.**
FeCoPdIrPt@GO	1 M KOH	42	82	[4]
HEI	1 M KOH	88.2	40.1	[5]
HEMP	1 M KOH	136	85.5	[6]
HF-HEA$_{a2}$	0.5 M H_2SO_4	73	39.7	[7]
$Ni_{20}Fe_{20}Mo_{10}Co_{35}Cr_{15}$	1 M KOH	172	66	[8]
$RhSe_2$	1 M KOH	81.6	96	[9]
$Ir_{25}Ni_{33}Ta_{42}$	0.5 M H_2SO_4	99	35	[10]
Pt_1/N-C	1 M KOH	46	36.8	[11]
Li-PPS ND	0.5 M H_2SO_4	91	29	[12]
PtNi-O/C	1 M KOH	39.8	78.8	[13]
Ru_1CoP/CDs-1000	1 M KOH	51	73.4	[14]
PdCu-B_2 NWs	1 M KOH	154.3	122	[15]
Ru-MoS_2/CNT	1 M KOH	50	62	[16]
S-M-5Pt	1 M KOH	62	78	[17]
$Ru_{0.10}$@2H-MoS_2	1 M KOH	51	64.9	[18]
**FeCoNiRu HEA**	**1 M KOH**	**40**	**84**	**This work**

Table S2. Overpotential, Tafel slope, charge transfer resistance, and double layer capacitance for as-prepared electrocatalysts for HER. The parameter of overpotential, Tafel slope, charge transfer resistance, and electrochemical double-layer capacitance for FeCoNiRu-X (X = 350, 400, 450, and 500) and the precious metal electrocatalysts for HER.

**catalysts**	**η_{10} (mV)**	**Tafel slope (mV dec^{-1})**	**R_{ct} (Ω)**	**C_{dl} (mF cm^{-2})**
FeCoNiRu-350	122	161	7.27	4.74
FeCoNiRu-400	61	120	4.58	13.03
FeCoNiRu-450	40	84	4.21	31.48
FeCoNiRu-500	117	122	6.31	9.24
Pt/C	22	55	5.76	-
RuO_2	87	191	9.25	-

Table S3. Corresponding parameter of EIS for HER. The parameter of R_s, R_o, and R_{ct} for FeCoNiRu-X (X = 350, 400, 450, and 500) and the noble metal electrocatalysts for HER.

**catalysts**	**R_s (Ω)**	**R_o (Ω)**	**R_{ct} (Ω)**
FeCoNiRu-350	1.27	0.124	7.27
FeCoNiRu-400	1.28	0.200	4.58
FeCoNiRu-450	1.21	0.094	4.21
FeCoNiRu-500	1.02	0.843	6.31
Pt/C	2.30	0.194	5.76
RuO_2	2.52	1.840	9.25

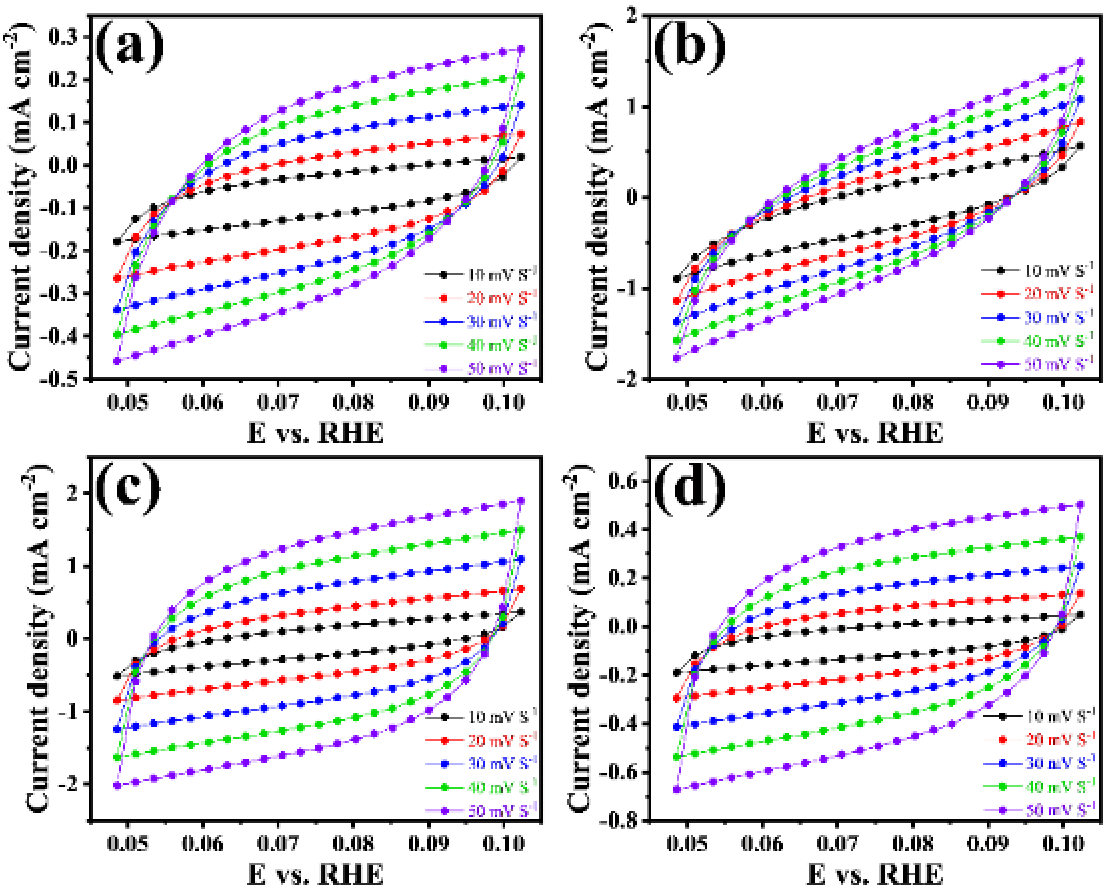

Figure S9 ECSA of FeCoNiRu-X (X = 350, 400, 450, and 500). CV curves of FeCoNiRu-350 (a), FeCoNiRu-400 (b), FeCoNiRu-450 (c), and FeCoNiRu-500 (d) at different scan rates.

Table S4. The values of TOFs. The values of TOFs of FeCoNiRu-450, the commercial Pt/C electrocatalyst, and the commercial RuO_2 electrocatalyst for HER and OER.

**catalysts**	**TOFs for HER @-0.1 V vs. RHE (S^{-1})**	**TOFs for OER @1.56 V vs. RHE (S^{-1})**
FeCoNiRu-450	0.046	0.084
Pt/C	0.047	0.003
RuO_2	0.022	0.068

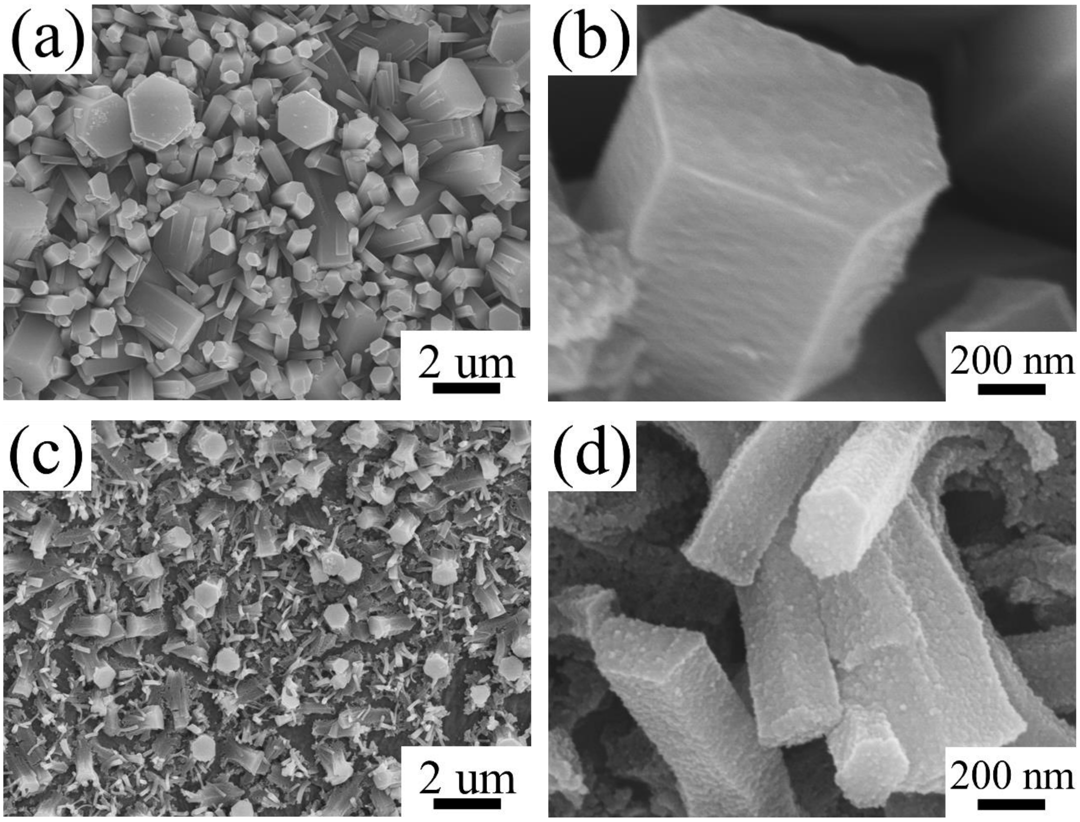

Figure S10 Morphological characterization. Low and high magnification FESEM images of unary Co-MOFs before (a, b) and after pyrolysis (c, d).

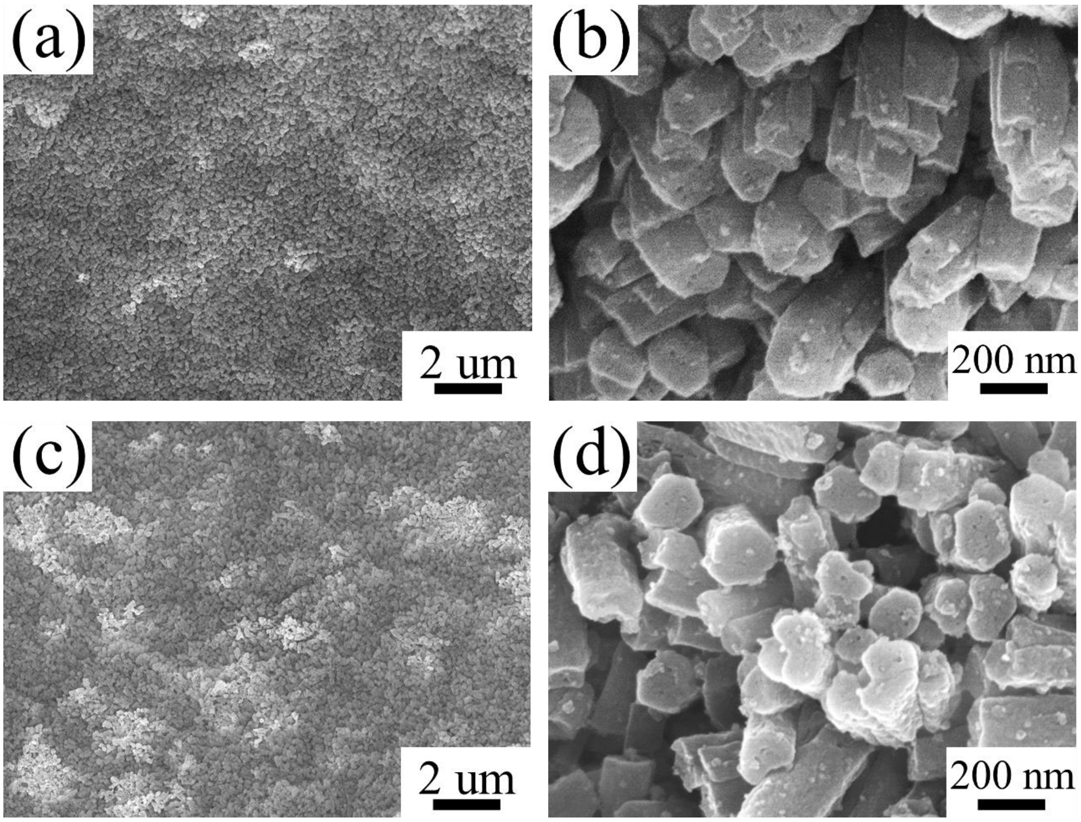

Figure S11 Morphological characterization. Low and high magnification FESEM images of binary FeCo-MOFs before (a, b) and after pyrolysis (c, d).

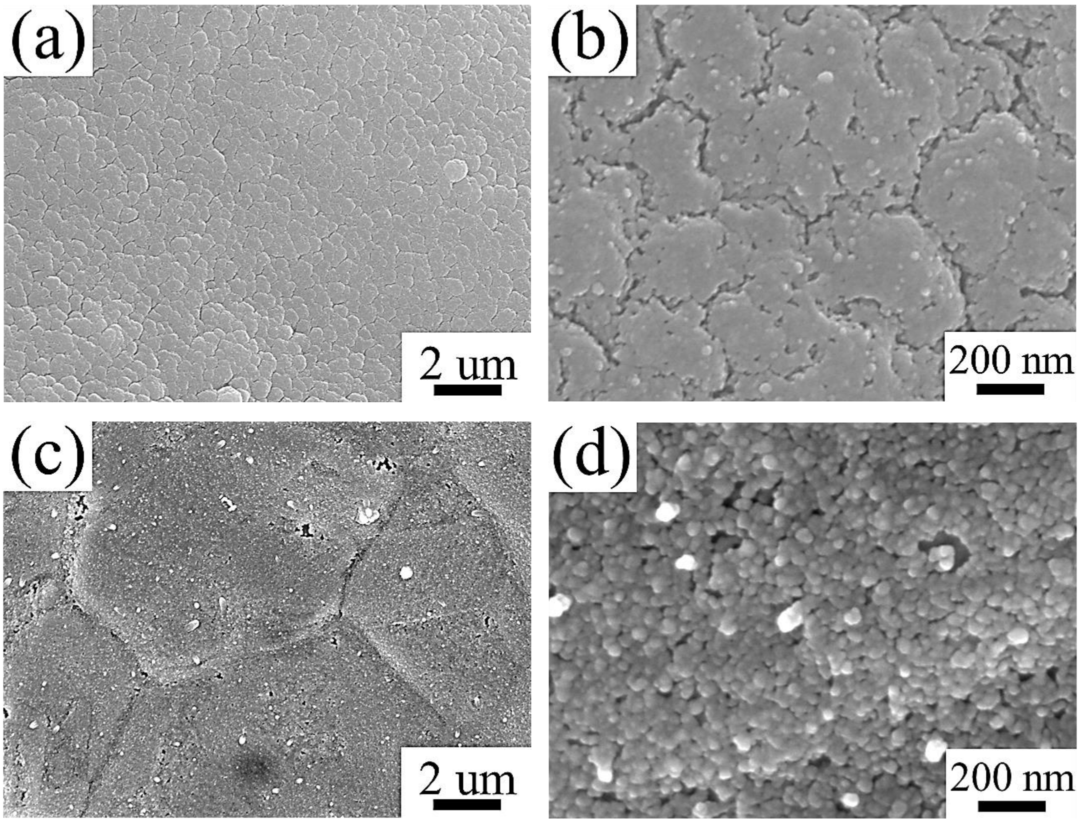

Figure S12 Morphological characterization. Low and high magnification FESEM images of ternary FeCoNi-MOFs before (a, b) and after pyrolysis (c, d).

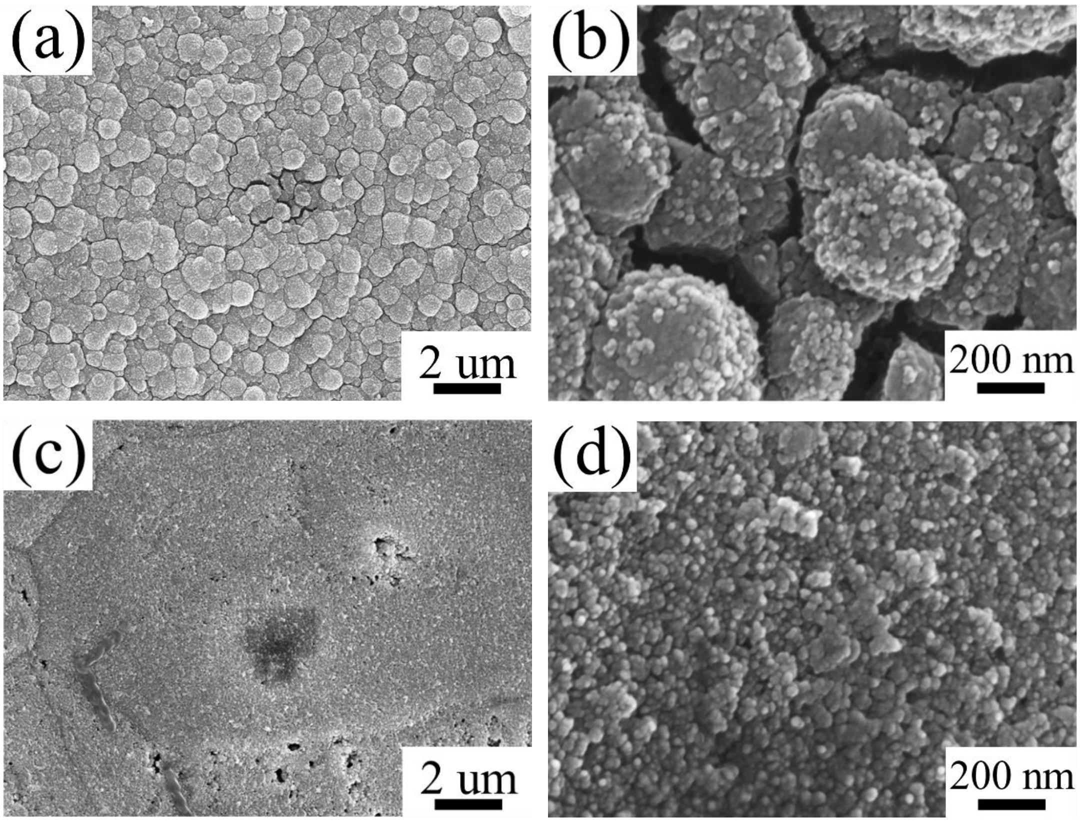

Figure S13 Morphological characterization. Low and high magnification FESEM images of ternary FeCoRu-MOFs before (a, b) and after pyrolysis (c, d).

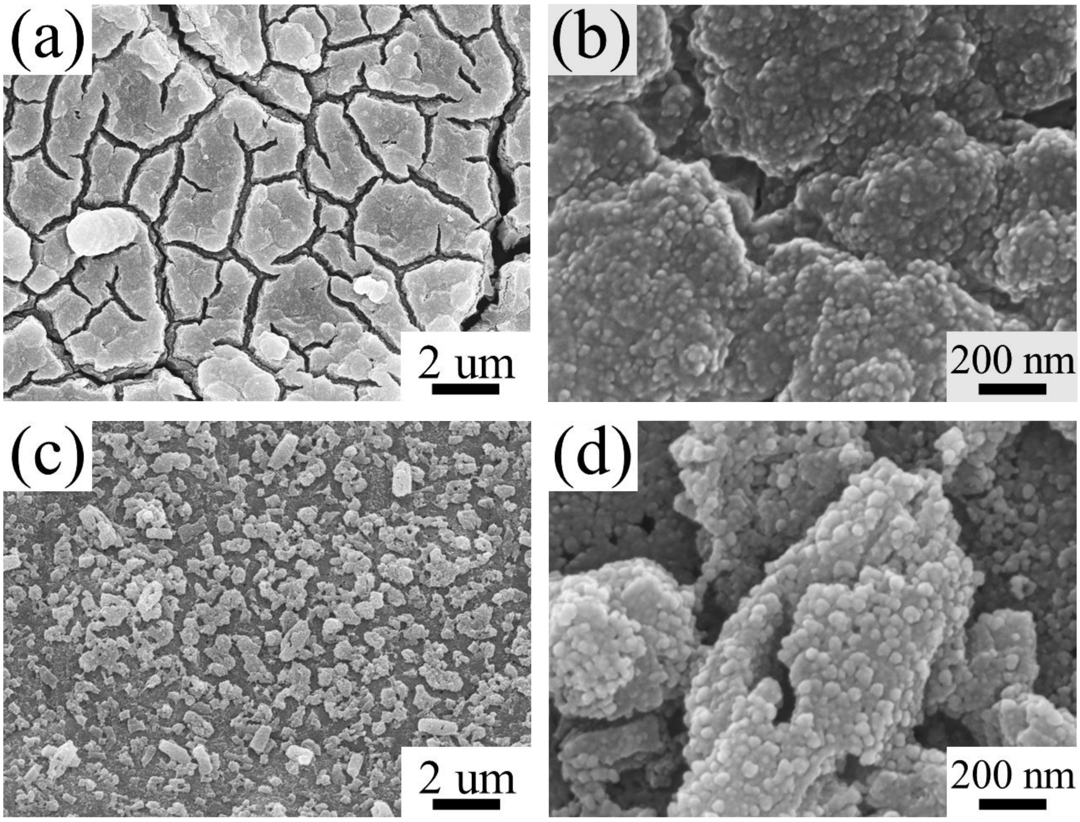

Figure S14 Morphological characterization. Low and high magnification FESEM images of ternary FeNiRu-MOFs before (a, b) and after pyrolysis (c, d).

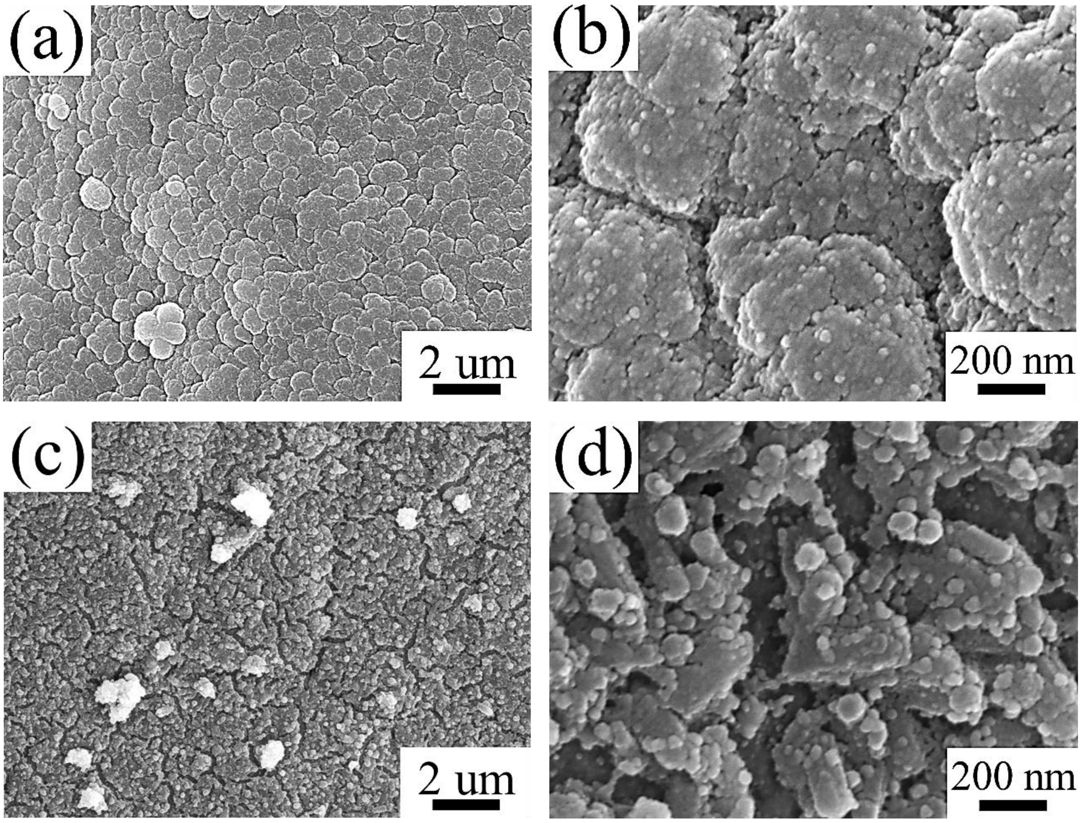

Figure S15 Morphological characterization. Low and high magnification FESEM images of ternary CoNiRu-MOFs before (a, b) and after pyrolysis (c, d).

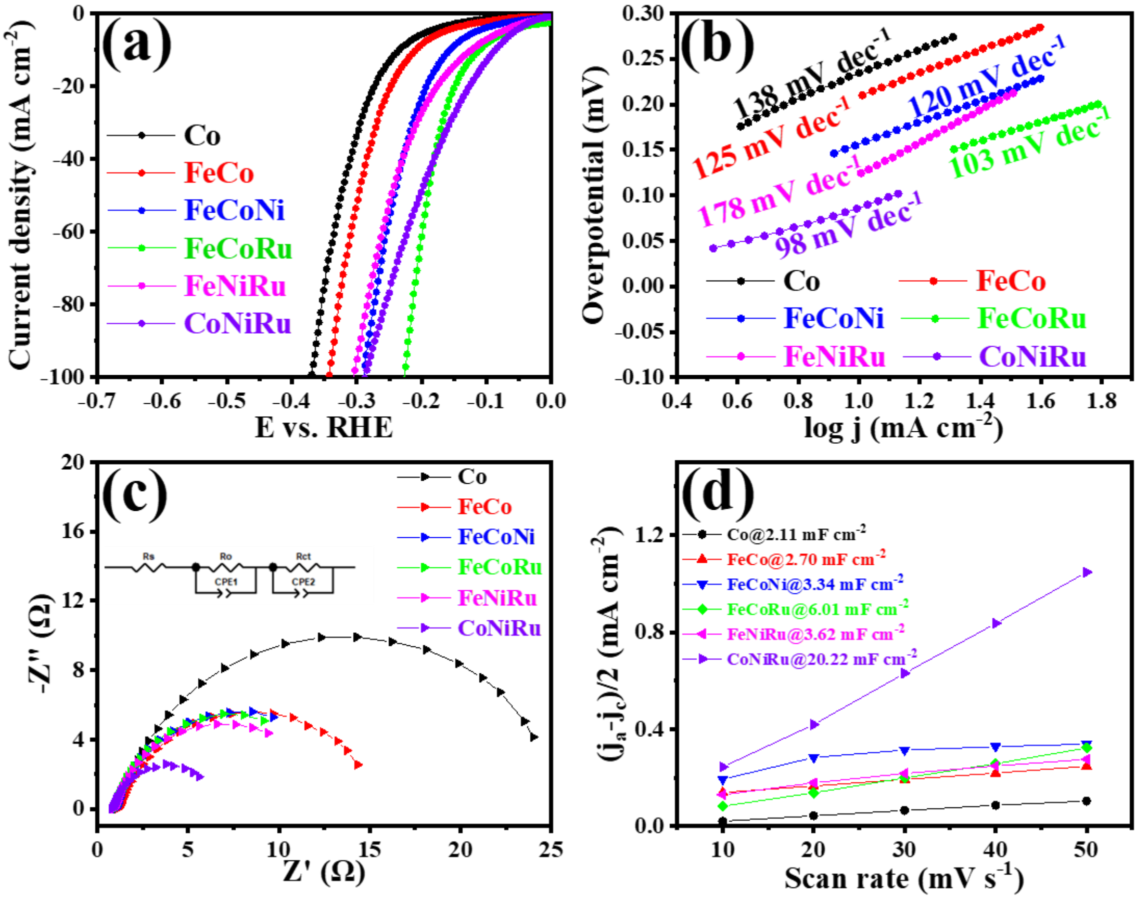

Figure S16 Electrocatalytic performance evaluation of unary Co, binary FeCo, ternary FeCoNi, FeCoRu, FeNiRu, and CoNiRu for HER. (a) LSV; (b) Tafel; (c) EIS; and (d) ECSA.

Table S5. Overpotential, Tafel slope, charge transfer resistance, and double layer capacitance for as-prepared electrocatalysts for HER. The parameter of overpotential, Tafel slope, charge transfer resistance, and electrochemical double-layer capacitance for as-prepared unary, binary, and ternary electrocatalytic materials for HER.

catalysts	η_{10} (mV)	Tafel slope (mV dec^{-1})	R_{ct} (Ω)	C_{dl} (mF cm^{-2})
Co	235	138	24.6	2.11
FeCo	209	125	13.3	2.70
FeCoNi	157	120	12.0	3.34
FeCoRu	111	103	8.9	6.01
FeNiRu	124	178	11.4	3.62
CoNiRu	87	98	5.57	20.22

Table S6. Corresponding parameters of EIS for HER. The parameter of R_s, R_o, and R_{ct} for as-prepared unary, binary, and ternary electrocatalytic materials for HER.

**catalysts**	**R_s (Ω)**	**R_o (Ω)**	**R_{ct} (Ω)**
Co	0.99	0.48	24.6
FeCo	1.14	1.20	13.3
FeCoNi	0.99	0.91	12.0
FeCoRu	0.91	5.2	8.9
FeNiRu	0.89	0.21	11.4
CoNiRu	0.81	0.26	5.57

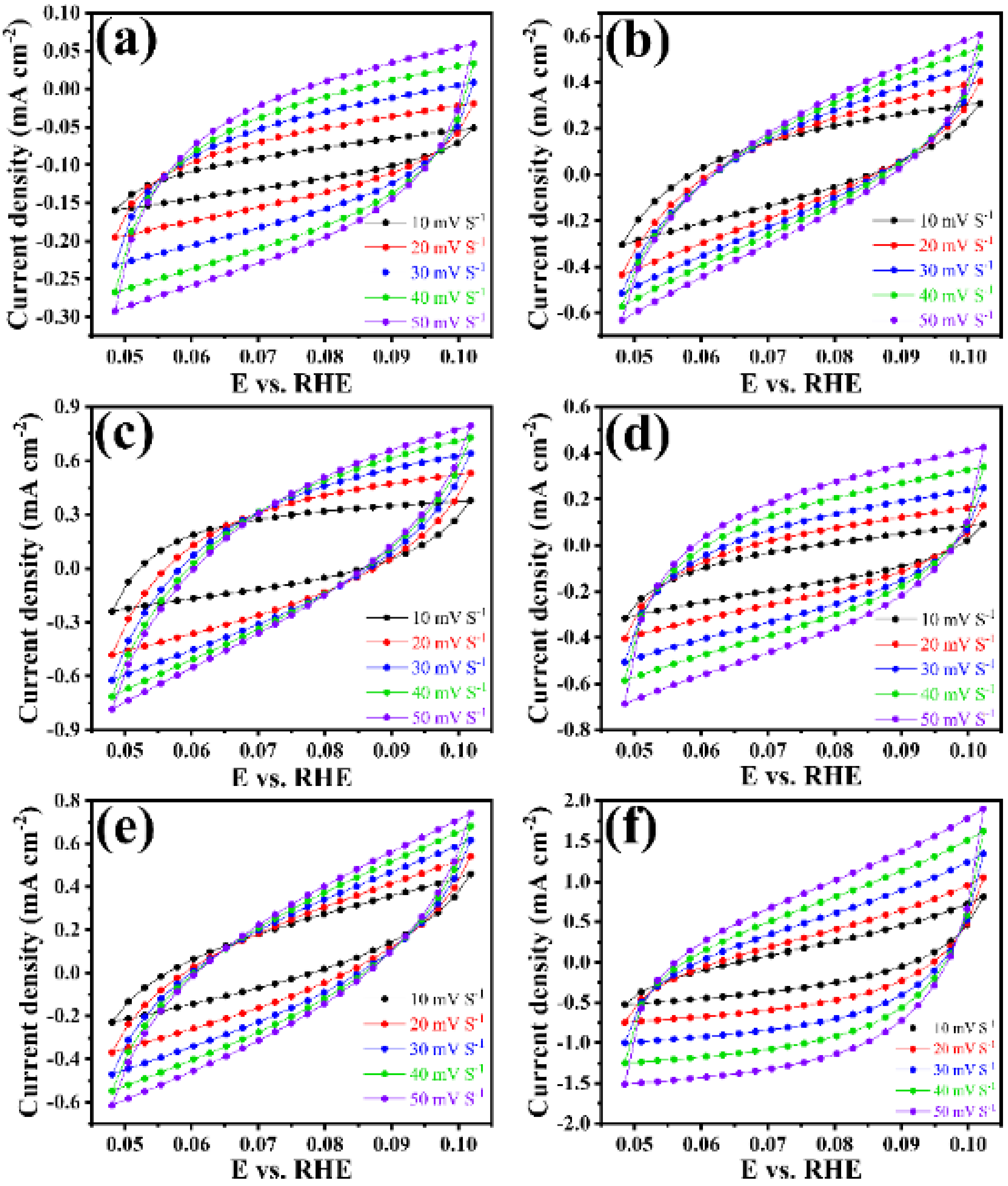

Figure S17 ECSA of unary, binary, and ternary electrocatalytic materials. CV curves of (a) uanry Co; (b) binary FeCo; (c) ternary FeCoNi; (d) FeCoRu; (e) FeNiRu; and (f) CoNiRu at differernt scan rates.

Table S7. Comparison of OER performance of FeCoNiRu-450 catalyst with recently reported HEAs and other noble metal-based OER electrocatalysts.

Materials	Electrolyte (KOH)	η_{10} (mV)	Tafel slope (mV dec^{-1})	Ref.
$K_{0.8}Na_{0.2}(MgMnFeCoNi)F_3$	1 M KOH	314	55	[19]
FeCoNiCuPd	1 M KOH	390	96	[20]
$La(CrMnFeCo_2Ni)O_3$	1 M KOH	325	51.2	[21]
MnFeCoNiCu	1 M KOH	263	43	[22]
np-AlFeCoNiMo	1 M KOH	240	46	[23]
SA-PtCoF	1 M KOH	308	68	[24]
30% Pt/$LiCoO_2$	1 M KOH	285	46.8	[25]
Ag@$Co(OH)_x$/CC	1 M KOH	250	76	[26]
$FeCoNiO_x$@IrPt HPNP	1 M KOH	240	34	[27]
RhCu NTs	1 M KOH	315	86	[28]
Co_3O_4-Ag@B	1 M KOH	270	62	[29]
Pt/Co-LDH	1 M KOH	265	63	[30]
ex-Ir-$Ni(OH)_2$	1 M KOH	270	45.2	[31]
Ir-C≡	1 M KOH	300	37	[32]
Ir@N-G-750	1 M KOH	270	62	[33]
**FeCoNiRu HEA**	**1 M KOH**	**243**	**45**	**This work**

Table S8. Overpotential, Tafel slope, and charge transfer resistance for as-prepared electrocatalysts for OER. The parameter of overpotential, Tafel slope, and charge transfer resistance, for FeCoNiRu-X (X = 350, 400, 450, and 500) electrocatalytic materials and the precious metal electrocatalysts for OER.

**catalysts**	**η_{10} (mV)**	**Tafel slope (mV dec^{-1})**	**R_{ct} (Ω)**
FeCoNiRu-350	304	103	5.34
FeCoNiRu-400	269	50	3.97
FeCoNiRu-450	243	45	3.48
FeCoNiRu-500	276	60	4.85
Pt/C	346	132	7.13
RuO_2	258	79	5.87

Table S9. Corresponding parameters of EIS for OER. The parameter of R_s, R_o, and R_{ct} for FeCoNiRu-X (X = 350, 400, 450, and 500) electrocatalytic materials and the noble metal electrocatalysts for OER.

**catalysts**	**R_s (Ω)**	**R_o (Ω)**	**R_{ct} (Ω)**
FeCoNiRu-350	1.18	0.511	5.34
FeCoNiRu-400	1.05	0.704	3.97
FeCoNiRu-450	1.02	0.491	3.48
FeCoNiRu-500	1.11	0.542	4.85
Pt/C	8.66	2.32	7.13
RuO_2	3.28	0.119	5.87

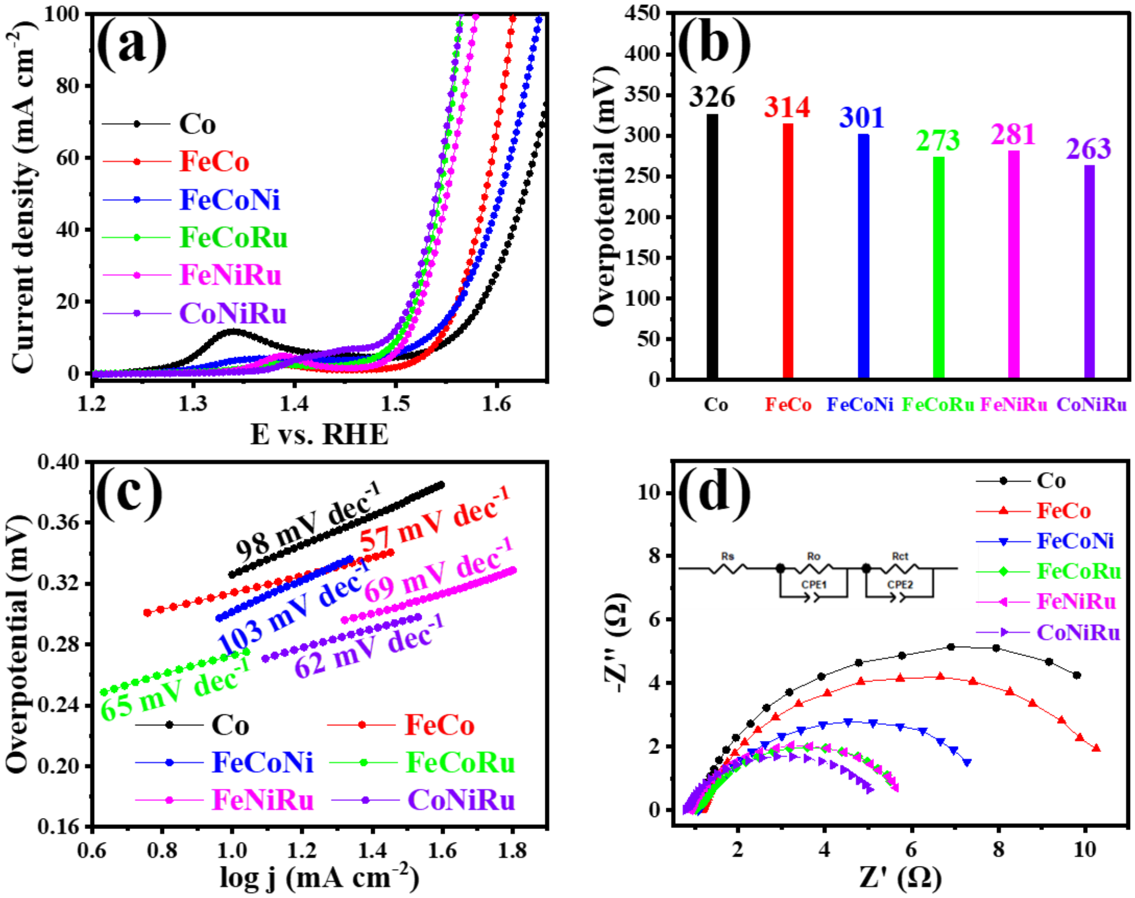

Figure S18 Electrocatalytic performance evaluation of unary Co, binary FeCo, ternary FeCoNi, FeCoRu, FeNiRu, and CoNiRu for OER. (a) LSV; (b) Overpotential; (c) Tafel; and (d) EIS.

Table S10. Overpotential, Tafel slope, and charge transfer resistance for as-prepared electrocatalysts for OER. The parameter of overpotential, Tafel slope, and charge transfer resistance for as-prepared unary, binary, and ternary electrocatalytic materials for OER.

**catalysts**	**η_{10} (mV)**	**Tafel slope (mV dec^{-1})**	**R_{ct} (Ω)**
Co	326	98	11.3
FeCo	314	57	9.28
FeCoNi	301	103	6.35
FeCoRu	273	65	4.53
FeNiRu	281	69	4.88
CoNiRu	263	62	4.34

Table S11. Corresponding parameters of EIS for OER. The parameter of R_s, R_o, and R_{ct} for as-prepared unary, binary, and ternary electrocatalytic materials for OER.

**catalysts**	**R_s (Ω)**	**R_o (Ω)**	**R_{ct} (Ω)**
Co	1.00	0.63	11.3
FeCo	1.14	0.48	9.28
FeCoNi	1.07	0.68	6.35
FeCoRu	1.01	0.33	4.53
FeNiRu	0.88	0.17	4.88
CoNiRu	0.78	0.08	4.34

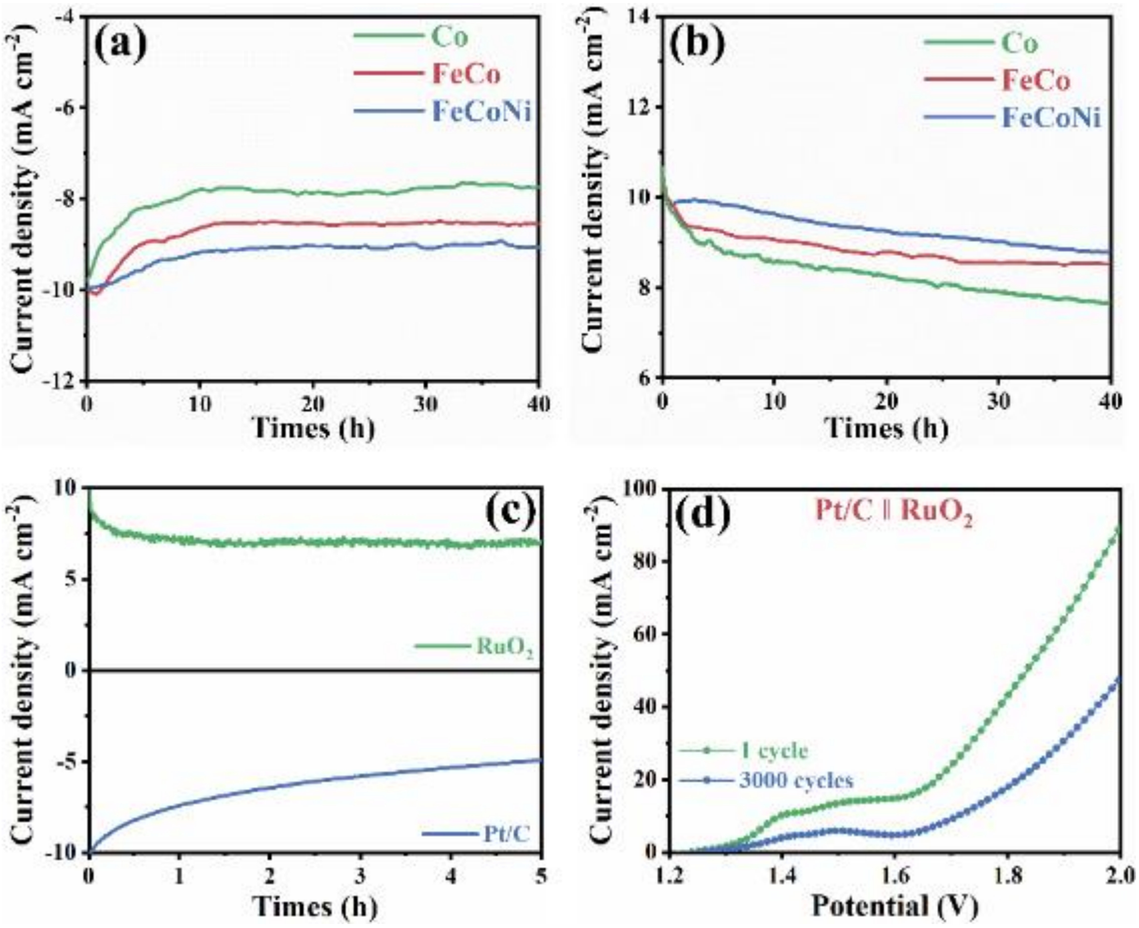

Figure S19 Stability test. Stability of ternary FeCoNi alloy, binary FeCo alloy, and unary Co for HER (a) and OER (b); Current density-time curve of Pt/C for HER and RuO_2 for OER (c); LSV of Pt/C ‖ RuO_2 before and after 3000 cycles.

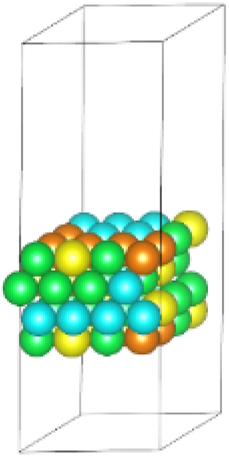

Figure S20 DFT calculations. Schematic diagram shows stable distribution of elements in alloys.

Tabel S12 H_2O-adsorption free energy at the top sites of HEA.

Number of sites	Adsorption energy (eV)	Top sites	Number of sites	Adsorption energy (eV)	Top sites
1	-0.50643	Ru	9	-0.36423	Co
2	-0.48684	Fe	10	-0.33472	Co
3	-0.48159	Ru	11	-0.31167	Ni
4	-0.46670	Fe	12	-0.30852	Ni
5	-0.44436	Fe	13	-0.28482	Co
6	-0.39949	Fe	14	-0.27137	Ni
7	-0.38822	Fe	15	-0.27032	Ni
8	-0.38235	Fe	16	-0.26413	Ni

*The number of sites corresponds to the number labeled in Figure 4a.

Tabel S13 H-adsorption free energy at each hollow sites of HEAs.

Number of sites	Adsorption energy (eV)	Hollow sites	Number of sites	Adsorption energy (eV)	Hollow sites
1	-0.44748	Ru-Fe-Fe	17	-0.54183	Ni-Fe-Fe
2	-0.48755	Ru-Co-Fe	18	-0.54809	Ru-Ni-Fe
3	-0.49068	Co-Fe-Fe	19	-0.55036	Ni-Ni-Ni
4	-0.49367	Ni-Ni-Fe	20	-0.55800	Ru-Co-Fe
5	-0.50290	Ru-Ni-Co	21	-0.55970	Ni-Co-Fe
6	-0.50520	Ru-Ni-Co	22	-0.56043	Ni-Ni-Fe
7	-0.50801	Ru-Ni-Co	23	-0.56184	Fe-Fe-Fe
8	-0.51780	Ru-Ni-Ni	24	-0.56638	Ru-Co-Fe
9	-0.52142	Ni-Ni-Co	25	-0.56647	Ni-Fe-Fe
10	-0.52221	Co-Fe-Fe	26	-0.56657	Ni-Co-Fe
11	-0.52526	Ru-Ni-Co	27	-0.58209	Co-Fe-Fe
12	-0.52552	Ni-Ni-Ni	28	-0.58601	Fe-Fe-Fe
13	-0.53084	Ru-Ni-Fe	29	-0.58753	Co-Fe-Fe
14	-0.53167	Ni-Ni-Ni	30	-0.59064	Ni-Fe-Fe
15	-0.53516	Ni-Ni-Co	31	-0.60557	Co-Fe-Fe
16	-0.54023	Ru-Co-Fe	32	-0.62088	Co-Fe-Fe

*The number of sites corresponds to the number labeled in Figure 4b.

Tabel S14 OH-adsorption free energy at Ru top sites and each hollow sites of HEAs.

**Number of sites**	**Adsorption energy (eV)**	**Top/Hollow sites**	**Number of sites**	**Adsorption energy (eV)**	**Hollow sites**
**1**	**-1.14731**	**Ru (top)**	16	-1.79722	Ni-Fe-Fe
**2**	**-1.15975**	**Ru (top)**	17	-1.80042	Ru-Co-Fe
1	-1.34892	Ni-Ni-Ni	18	-1.81162	Ru-Co-Fe
2	-1.43609	Ni-Ni-Ni	19	-1.84198	Co-Fe-Fe
3	-1.46242	Ru-Ni-Co	20	-1.85607	Co-Fe-Fe
4	-1.50393	Ru-Ni-Co	21	-1.85727	Ru-Co-Fe
5	-1.53192	Ru-Ni-Ni	22	-1.85736	Ru-Ni-Fe
6	-1.54749	Ni-Ni-Co	23	-1.89671	Ni-Fe-Fe
7	-1.54898	Ru-Ni-Co	24	-1.92243	Co-Fe-Fe
8	-1.56775	Ni-Ni-Fe	25	-1.92776	Ni-Ni-Fe
9	-1.57347	Ni-Ni-Co	26	-1.93741	Ni-Fe-Fe
10	-1.60077	Ni-Ni-Ni	27	-1.96704	Co-Fe-Fe
11	-1.61440	Ru-Co-Fe	28	-1.99976	Fe-Fe-Fe
12	-1.65824	Ru-Ni-Co	29	-2.01186	Ni-Co-Fe
13	-1.69772	Ni-Co-Fe	30	-2.01190	Co-Fe-Fe
14	-1.70882	Ru-Ni-Fe	31	-2.01702	Co-Fe-Fe
15	-1.76876	Ru-Fe-Fe	32	-2.11321	Fe-Fe-Fe

*The number of sites corresponds to the number labeled in Figure 4f.

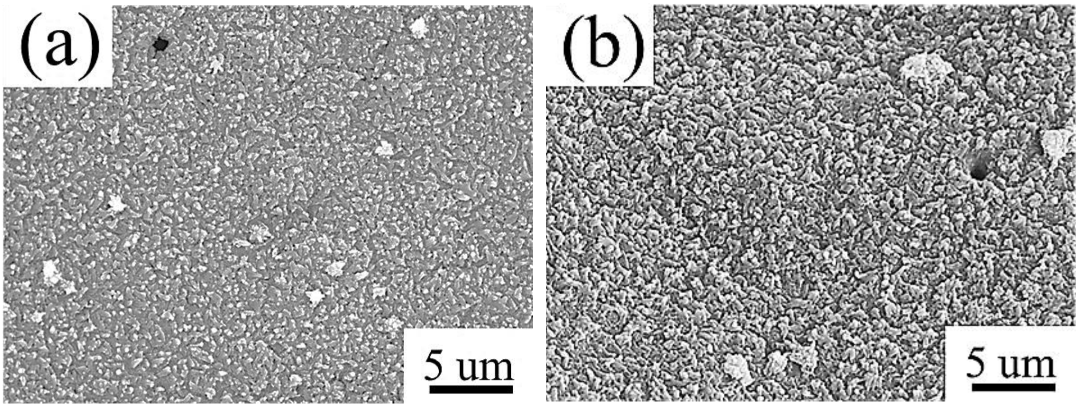

Figure S21 Morphological characterization. Low magnification FESEM images of FeCoNiRu-450 electrocatalysts after the HER LSV test (a) and stability test (b).

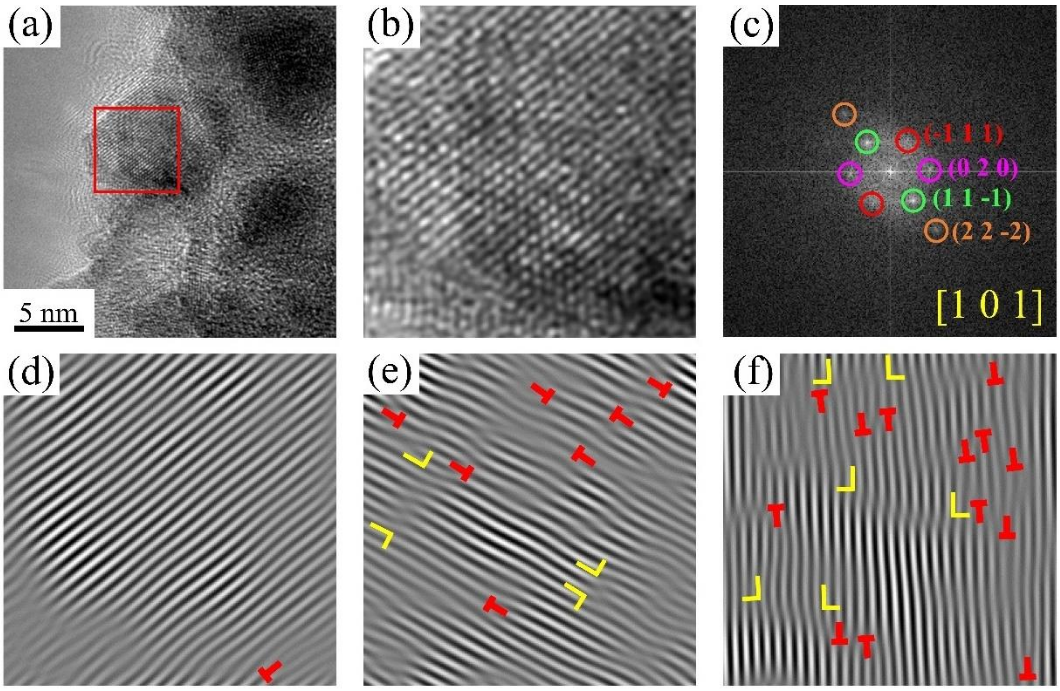

Figure S22 HRTEM analysis of the fully exposed FeCoNiRu-450 nanoparticle after the HER LSV test. (a) HRTEM; (b) zoom in HRTEM image taken from the red squared area marked in (a); (c) FFT pattern of (b); (d-f) IFFT along with (1 1 -1), (-1 1 1), and (0 2 0).

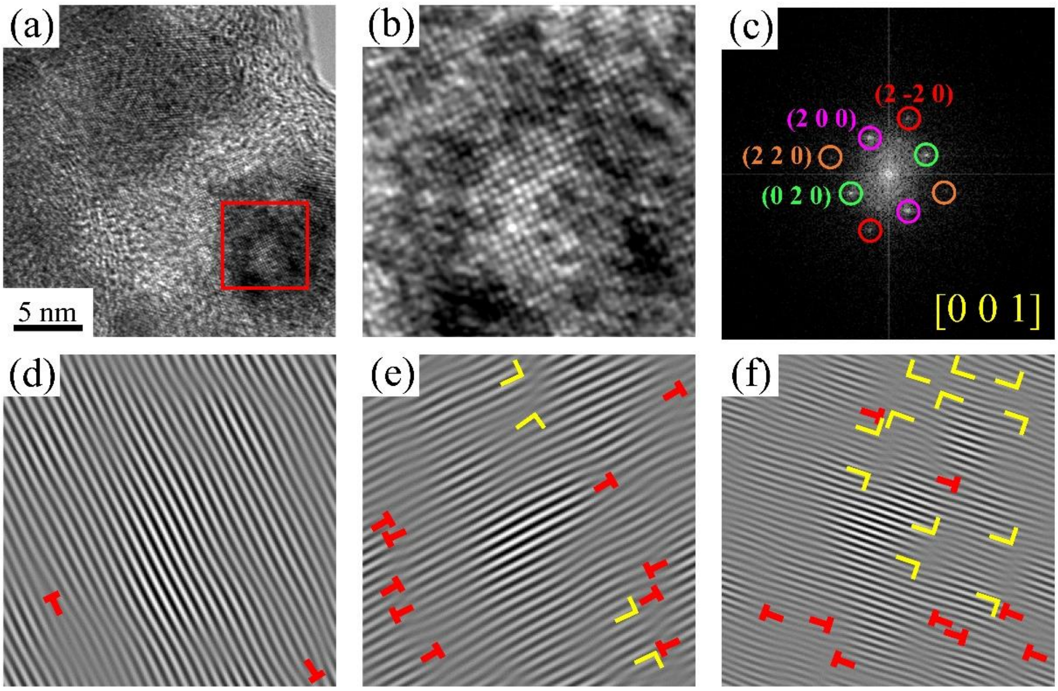

Figure S23 HRTEM analysis of the encapsulated by carbon layers FeCoNiRu-450 nanoparticle after the HER LSV test. (a) HRTEM; (b) zoom in HRTEM image taken from the red squared area marked in (a); (c) FFT pattern of (b); (d-f) IFFT along with (0 2 0), (2 0 0), and (2 -2 0).

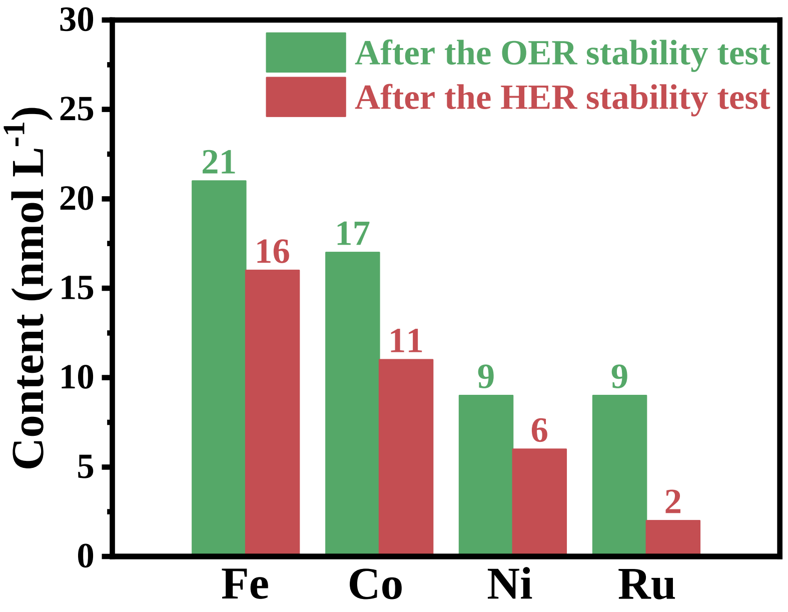

Figure S24 ICP-MS analysis. The content of each element in the electrolyte after the HER and OER stability test.

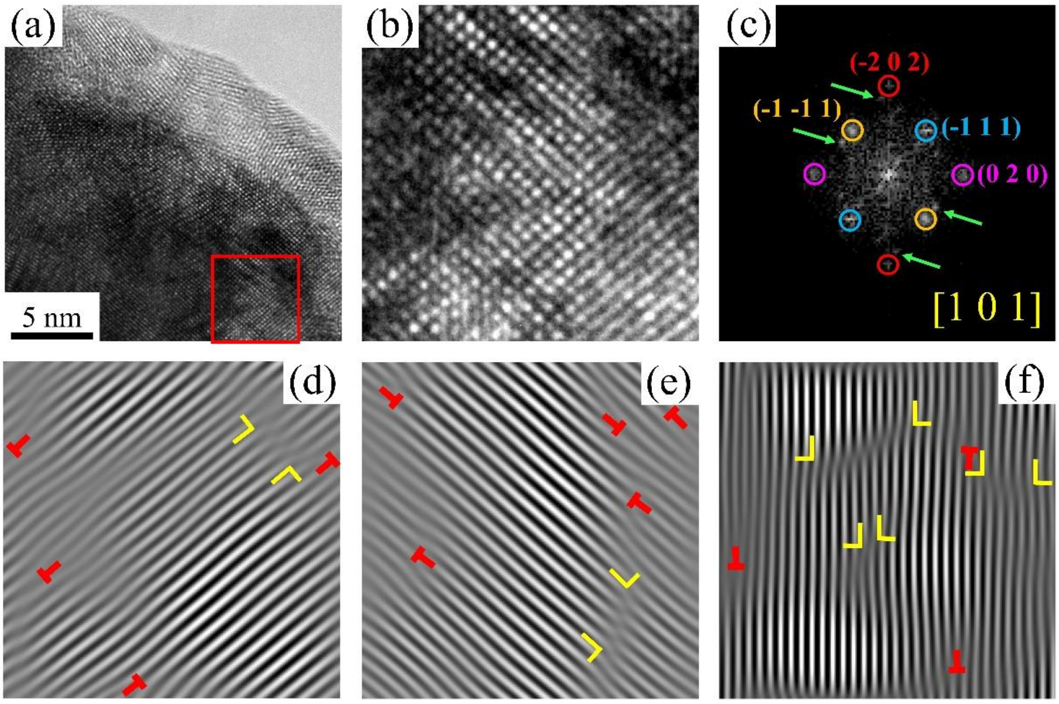

Figure S25 HRTEM analysis of the non-oxidized matrix of fully exposed FeCoNiRu-450 nanoparticle after the HER stability test. (a) HRTEM; (b) zoom in HRTEM image taken from the red squared area marked in (a); (c) FFT pattern of (b); (d-f) IFFT along with (-1 -1 1), (-1 1 1), and (0 2 0).

Other diffraction spots pointed out by the green arrow are attributed to the Moiré fringes generated by the overlapping of two nanoparticles.

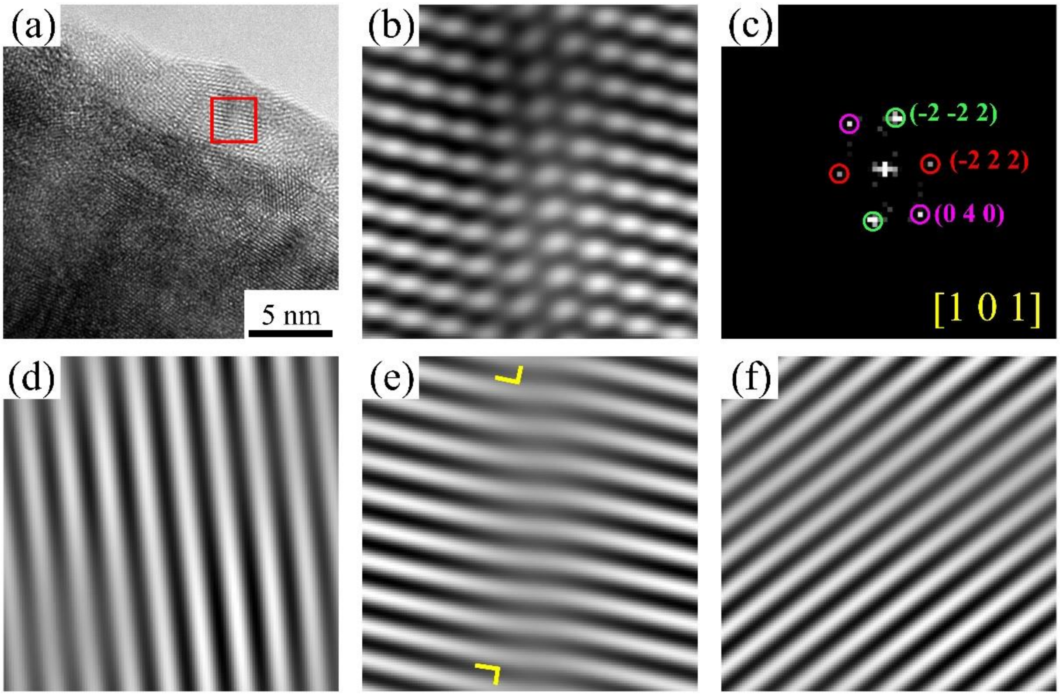

Figure S26 HRTEM analysis of the oxide layer of fully exposed FeCoNiRu-450 nanoparticle after the HER stability test. (a) HRTEM; (b) zoom in HRTEM image taken from the red squared area marked in (a); (c) FFT pattern of (b); (d-f) IFFT along with (-2 2 2), (-2 -2 2), and (0 4 0).

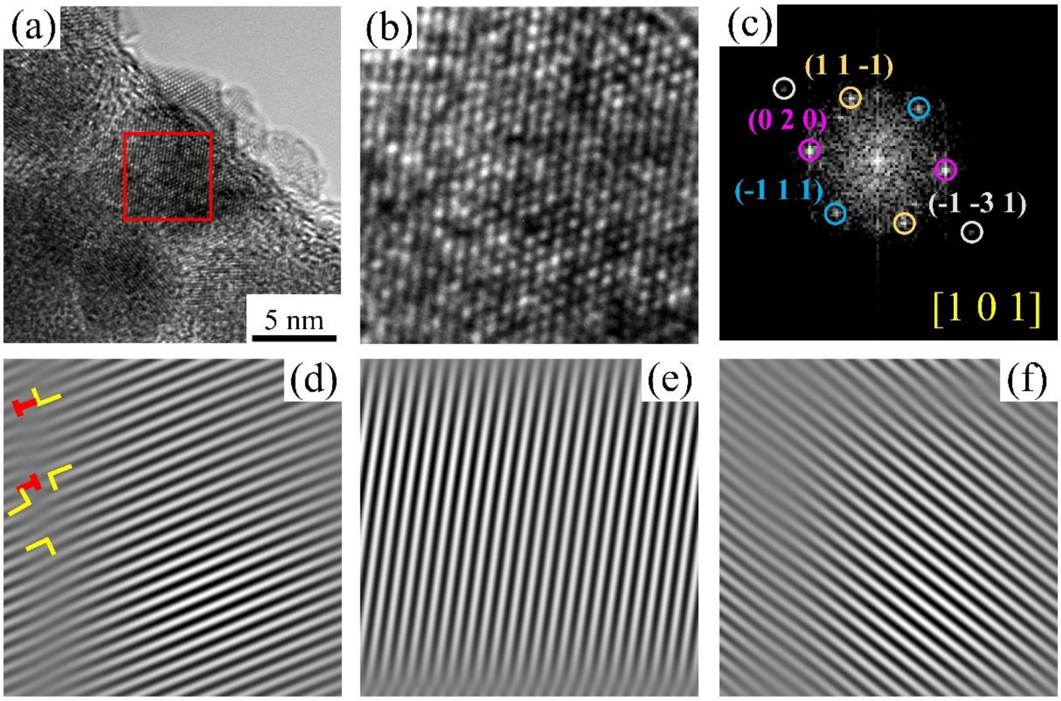

Figure S27 HRTEM analysis of the encapsulated by carbon layers FeCoNiRu-450 nanoparticle after the HER stability test. (a) HRTEM; (b) zoom in HRTEM image taken from the red squared area marked in (a); (c) FFT pattern of (b); (d-f) IFFT along with (1 1 -1), (0 2 0), and (-1 1 1).

Other diffraction spots are originated from the oxide under this nanoparticle.

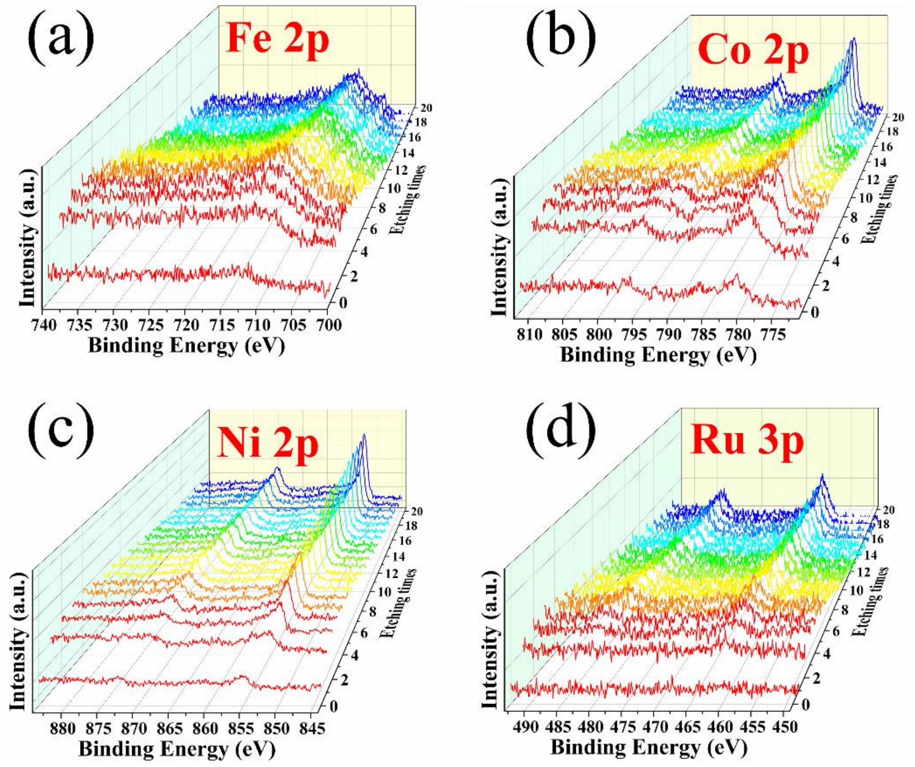

Figure S28 XPS compositional analysis of FeCoNiRu-450 nanoparticles after the HER stability test. (a-d) Fe 2p, Co 2p, Ni 2p, and Ru 3p spectra of FeCoNiRu-450 at different number of times of etching.

To understand the depth profile of the oxide layer, the etching treatment of the surface of the FeCoNiRu-450 electrocatalyst was carried out by small ion clusters at a slow speed 19 times each time of 10 seconds. Oxidation decays across the depth towards the matrix, which also proves the oxide formation on the surface of FeCoNiRu-450 nanoparticles after the HER stability process. All these XPS results are also consistent with the HRTEM analysis.

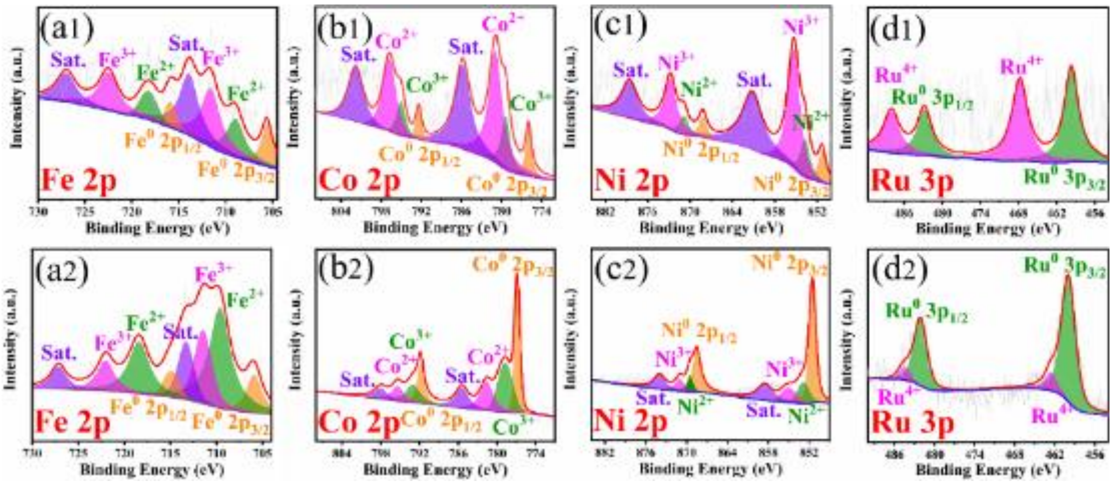

Figure S29 XPS compositional analysis of FeCoNiRu-450 nanoparticles after the HER stability test. High-resolution spectra of Fe, Co, Ni, and Ru before (a1-d1) and after (a2-d2) etching, respectively.

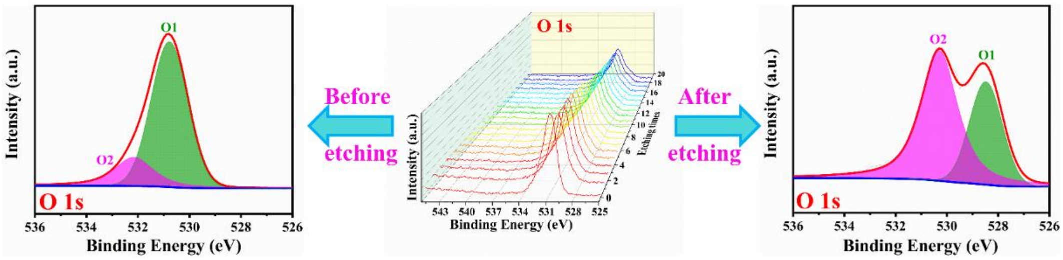

Figure S30 XPS compositional analysis of FeCoNiRu-450 nanoparticles after the HER stability test. O 1s spectra before and after etching process.

The high-resolution O 1s spectrum can be fitted into two peaks of O1 and O2, which are attributed to the lattice oxygen and surface-adsorbed oxygen-containing species (such as hydroxide or water), respectively.

Tabel S15 H_2O-adsorption free energy at each hollow sites of spinel oxide.

**Hollow sites (tet-oct-oct)**	**Adsorption energy (eV)**	**Hollow sites (tet-oct-oct)**	**Adsorption energy (eV)**
CoCoCo	-0.6698	CoNiCo	-0.7163
FeCoCo	-0.8929	CoNiRu	-0.5068
NiCoCo	-0.6268	CoRuCo	-0.6449
CoFeFe	-0.6310	FeFeCo	-0.8987
FeFeFe	-0.8697	FeFeNi	-0.8534
NiFeFe	-0.5029	FeFeRu	-0.8841
CoRuRu	-0.6662	FeNiCo	-0.9223
FeRuRu	-0.8754	FeNiRu	-0.9182
NiRuRu	-0.5653	FeRuCo	-0.8646
CoNiNi	-0.7165	NiFeCo	-0.5845
FeNiNi	-0.8894	NiFeNi	-0.6299
NiNiNi	-0.7131	NiFeRu	-0.5729
CoFeCo	-0.6777	NiNiCo	-0.7059
CoFeNi	-0.6689	NiNiRu	-0.6082
CoFeRu	-0.6534	NiRuCo	-0.6506

Tabel S16 H-adsorption free energy at each hollow sites of spinel oxide.

**Hollow sites (tet-oct-oct)**	**Adsorption energy (eV)**	**Hollow sites (tet-oct-oct)**	**Adsorption energy (eV)**
CoCoCo	0.9545	CoNiCo	0.9548
FeCoCo	0.5910	CoNiRu	0.9185
NiCoCo	1.4141	CoRuCo	0.9613
CoFeFe	0.9514	FeFeCo	0.6023
FeFeFe	0.5906	FeFeNi	0.6251
NiFeFe	1.4131	FeFeRu	0.5841
CoRuRu	0.9411	FeNiCo	0.5899
FeRuRu	0.5866	FeNiRu	0.5698
NiRuRu	1.4084	FeRuCo	0.6123
CoNiNi	0.9642	NiFeCo	1.4178
FeNiNi	0.6041	NiFeNi	1.4253
NiNiNi	1.4020	NiFeRu	1.3917
CoFeCo	0.9627	NiNiCo	1.4121
CoFeNi	0.9779	NiNiRu	1.3737
CoFeRu	0.9341	NiRuCo	1.4082

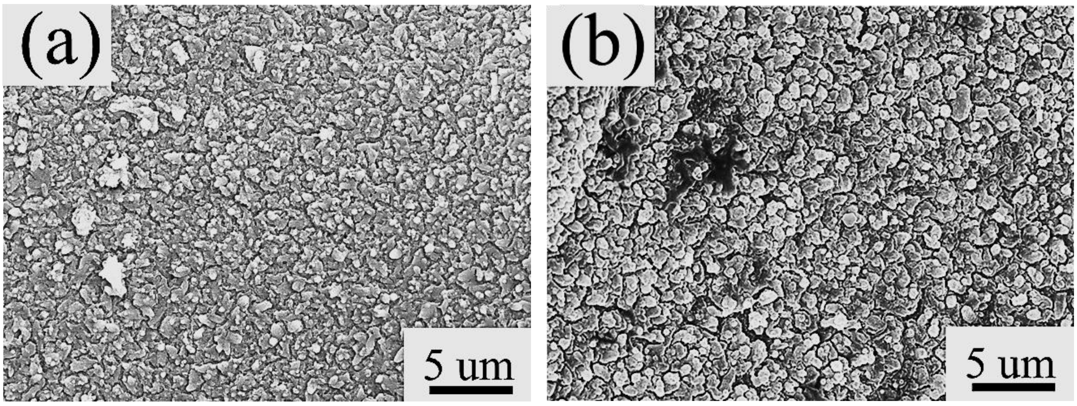

Figure S31 Morphological characterization. Low magnification FESEM images of FeCoNiRu-450 electrocatalysts after the OER LSV test (a) and stability test (b).

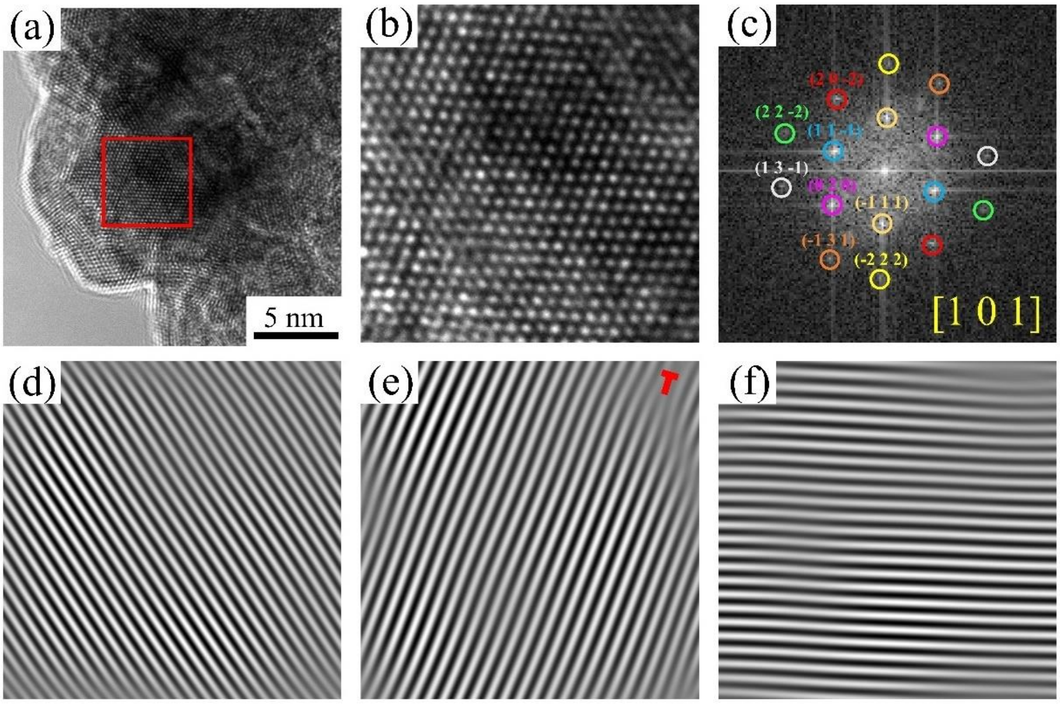

Figure S32 HRTEM analysis of the non-oxdized matrix of fully exposed FeCoNiRu-450 nanoparticle after the OER LSV test. (a) HRTEM; (b) zoom in HRTEM image taken from the red squared area marked in (a); (c) FFT pattern of (b); (d-f) IFFT along with (0 2 0), (1 1 -1), and (-1 1 1).

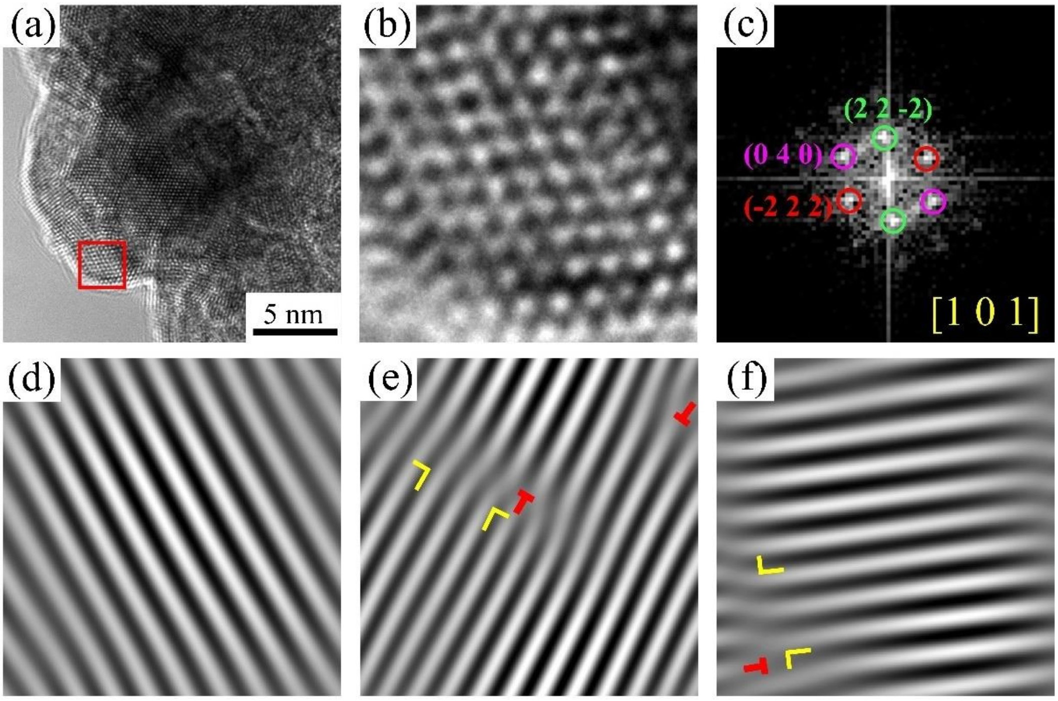

Figure S33 HRTEM analysis of the oxide layer of fully exposed FeCoNiRu-450 nanoparticle after the OER LSV test. (a) HRTEM; (b) zoom in HRTEM image taken from the red squared area marked in (a); (c) FFT pattern of (b); (d-f) IFFT along with (-2 2 2), (0 4 0), and (2 2 -2).

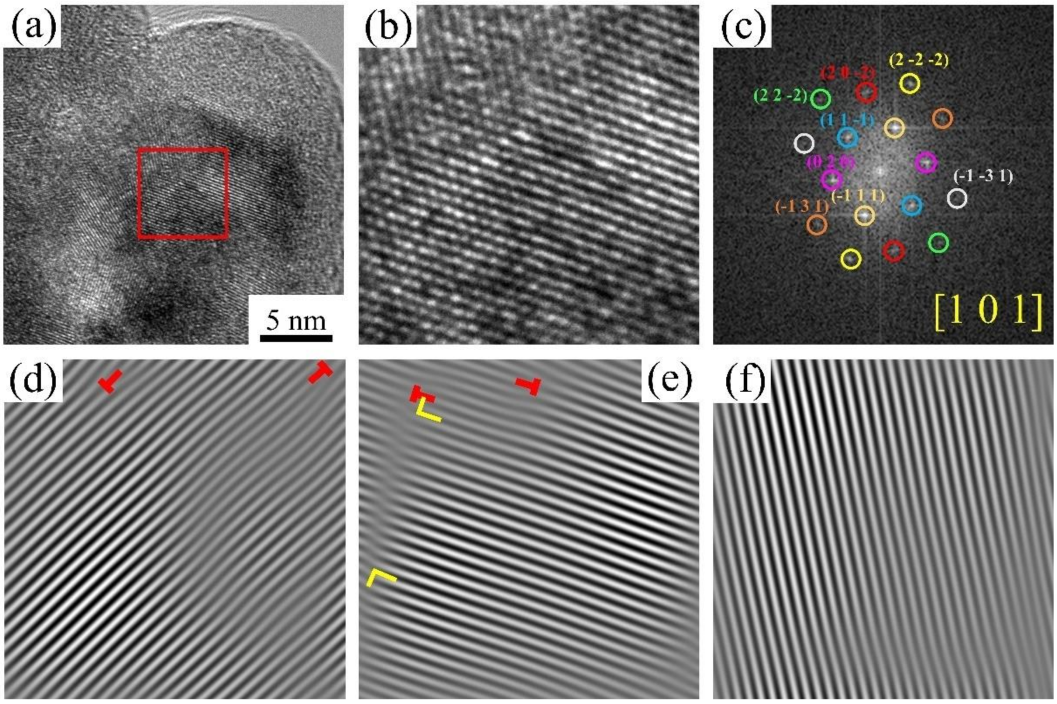

Figure S34 HRTEM analysis of the encapsulated by carbon layers FeCoNiRu-450 nanoparticle after the OER LSV test. (a) HRTEM; (b) zoom in HRTEM image taken from the red squared area marked in (a); (c) FFT pattern of (b); (d-f) IFFT along with (1 1 -1), (-1 1 1), and (0 2 0).

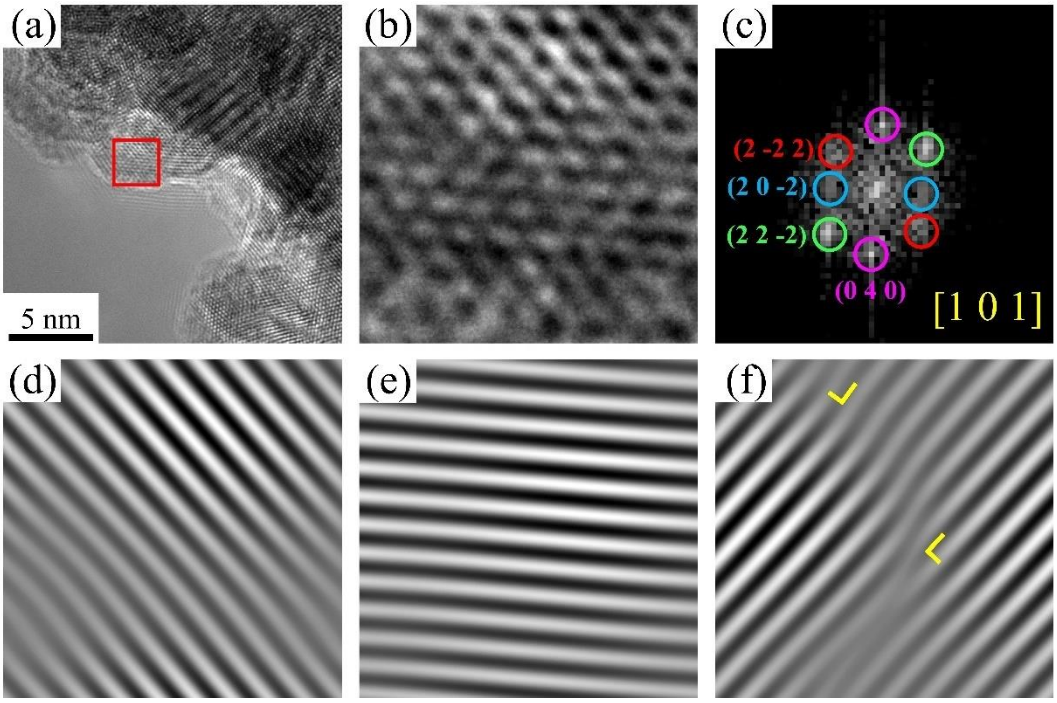

Figure S35 HRTEM analysis of the fully exposed FeCoNiRu-450 nanoparticle after the OER stability test. (a) HRTEM; (b) zoom in HRTEM image taken from the red squared area marked in (a); (c) FFT pattern of (b); (d-f) IFFT along with (2 2 -2), (0 4 0), and (2 -2 2).

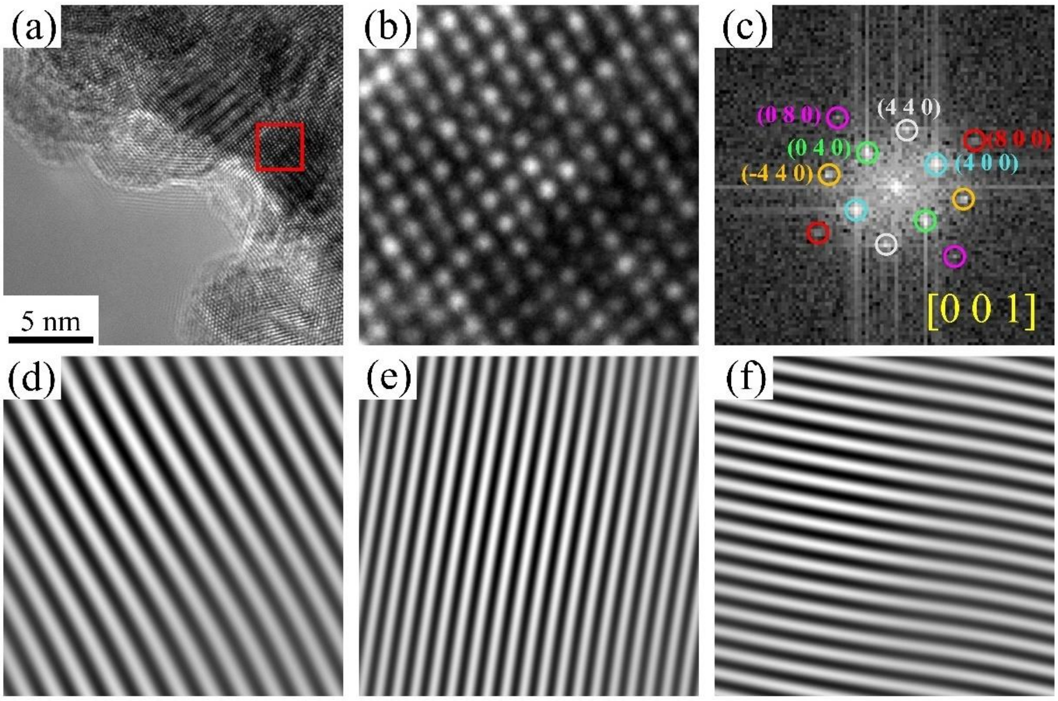

Figure S36 HRTEM analysis of the fully exposed FeCoNiRu-450 nanoparticle after the OER stability test. (a) HRTEM; (b) zoom in HRTEM image taken from the red squared area marked in (a); (c) FFT pattern of (b); (d-f) IFFT along with (4 0 0), (-4 4 0), and (4 4 0).

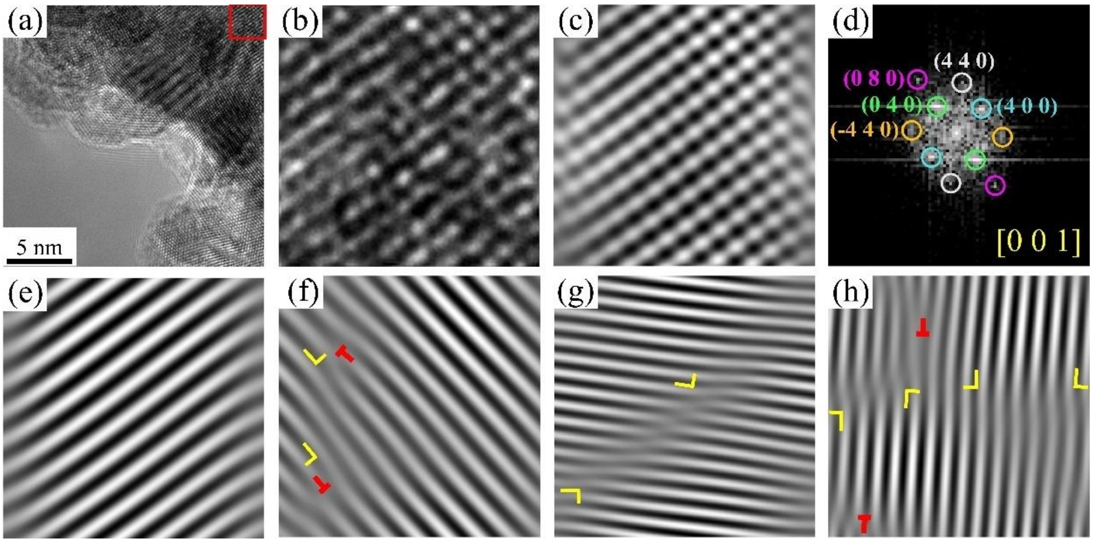

Figure S37 HRTEM analysis of the fully exposed FeCoNiRu-450 nanoparticle after the OER stability test. (a) HRTEM; (b) zoom in HRTEM image taken from the red squared area marked in (a); (c) IFFT image of (b); (d) FFT pattern of (b); (e-h) IFFT along with (0 4 0), (4 0 0), (4 4 0), and (-4 4 0).

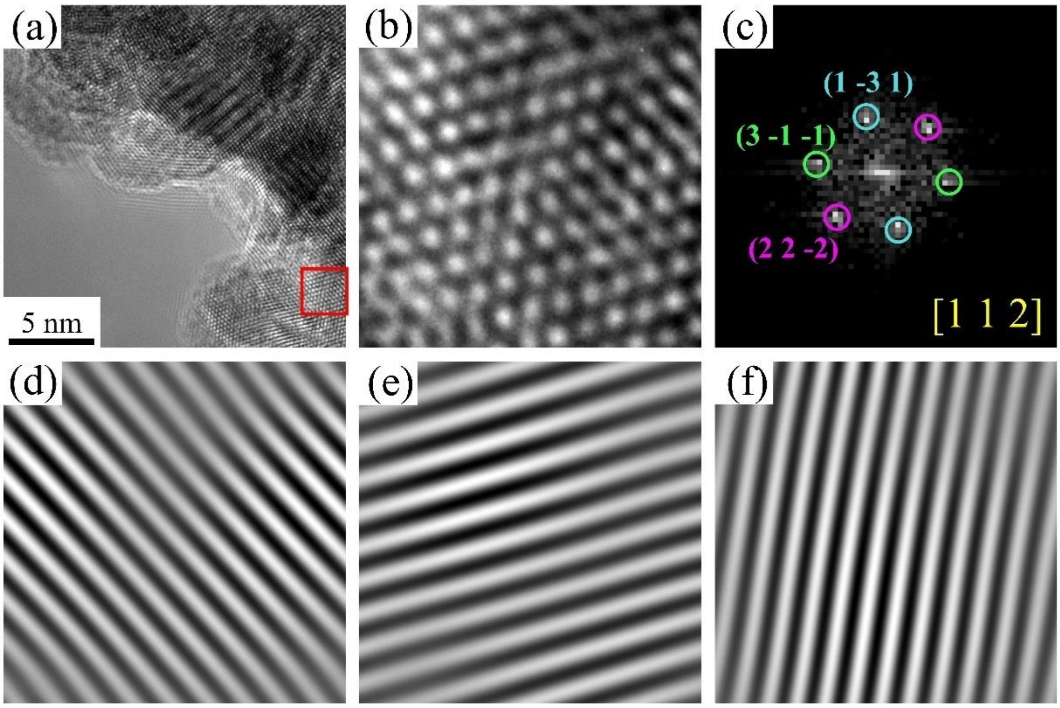

Figure S38 HRTEM analysis of the fully exposed FeCoNiRu-450 nanoparticle after the OER stability test. (a) HRTEM; (b) zoom in HRTEM image taken from the red squared area marked in (a); (c) FFT pattern of (b); (d-f) IFFT along with (2 2 -2), (1 -3 1), and (3 -1 -1).

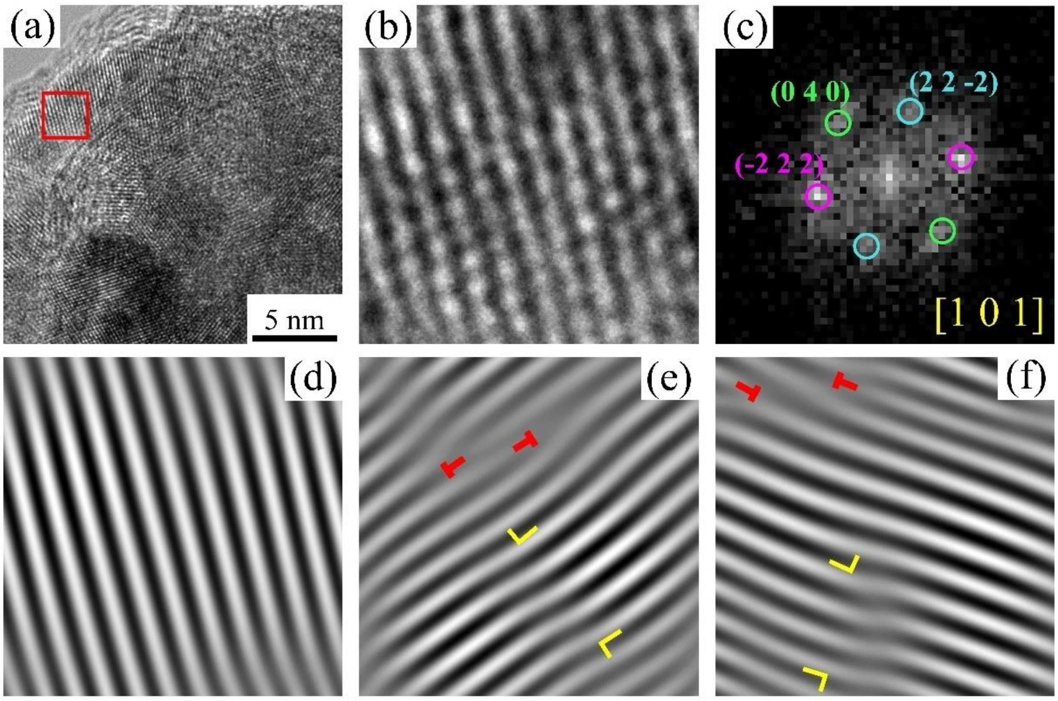

Figure S39 HRTEM analysis of the encapsulated by carbon layers FeCoNiRu-450 nanoparticle after the OER stability test. (a) HRTEM; (b) zoom in HRTEM image taken from the red squared area marked in (a); (c) FFT pattern of (b); (d-f) IFFT along with (-2 2 2), (0 4 0), and (2 2 -2).

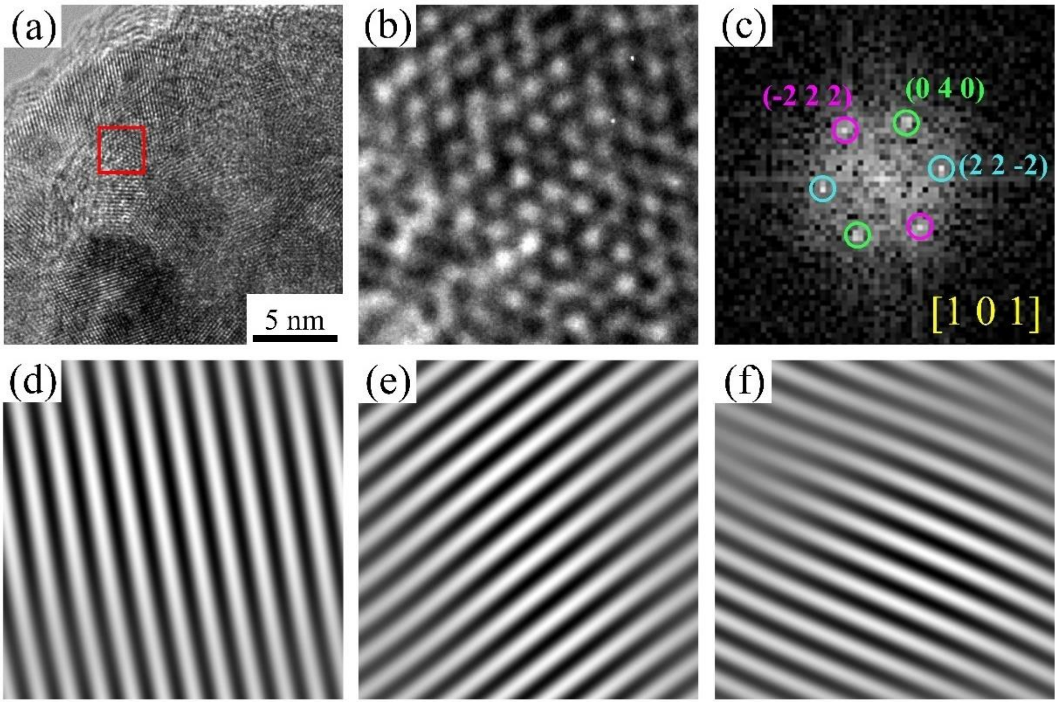

Figure S40 HRTEM analysis of the encapsulated by carbon layers FeCoNiRu-450 nanoparticle after the OER stability test. (a) HRTEM; (b) zoom in HRTEM image taken from the red squared area marked in (a); (c) FFT pattern of (b); (d-f) IFFT along with (2 2 -2), (-2 2 2), and (0 4 0).

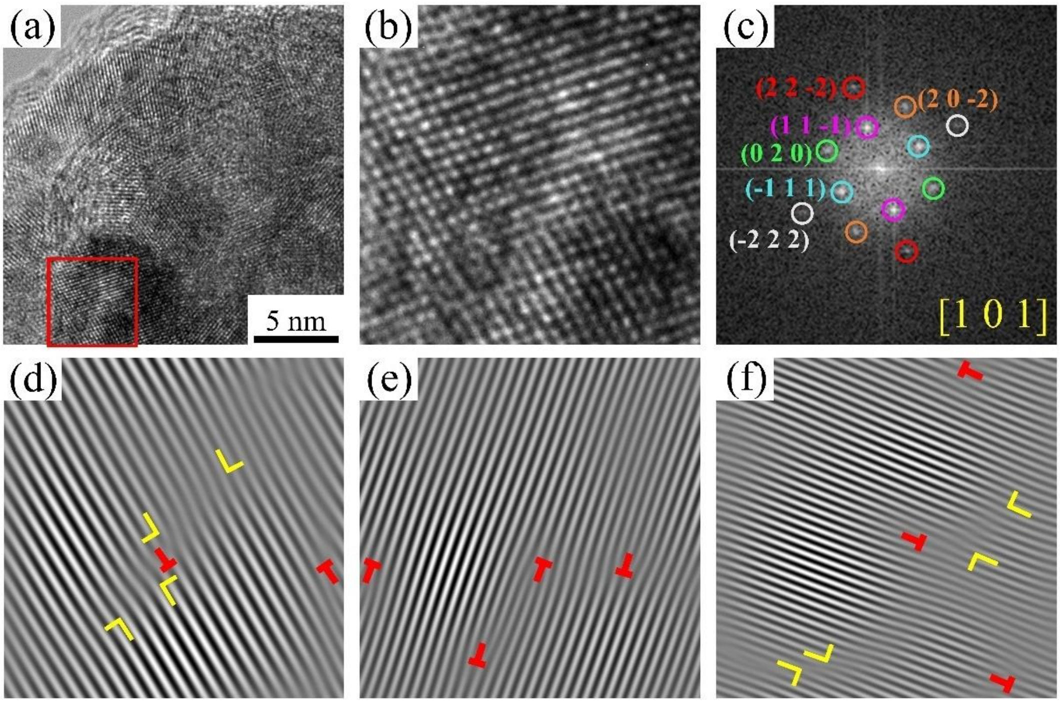

Figure S41 HRTEM analysis of the encapsulated by carbon layers FeCoNiRu-450 nanoparticle after the OER stability test. (a) HRTEM; (b) zoom in HRTEM image taken from the red squared area marked in (a); (c) FFT pattern of (b); (d-f) IFFT along with (-1 1 1), (0 2 0), and (2 0 -2).

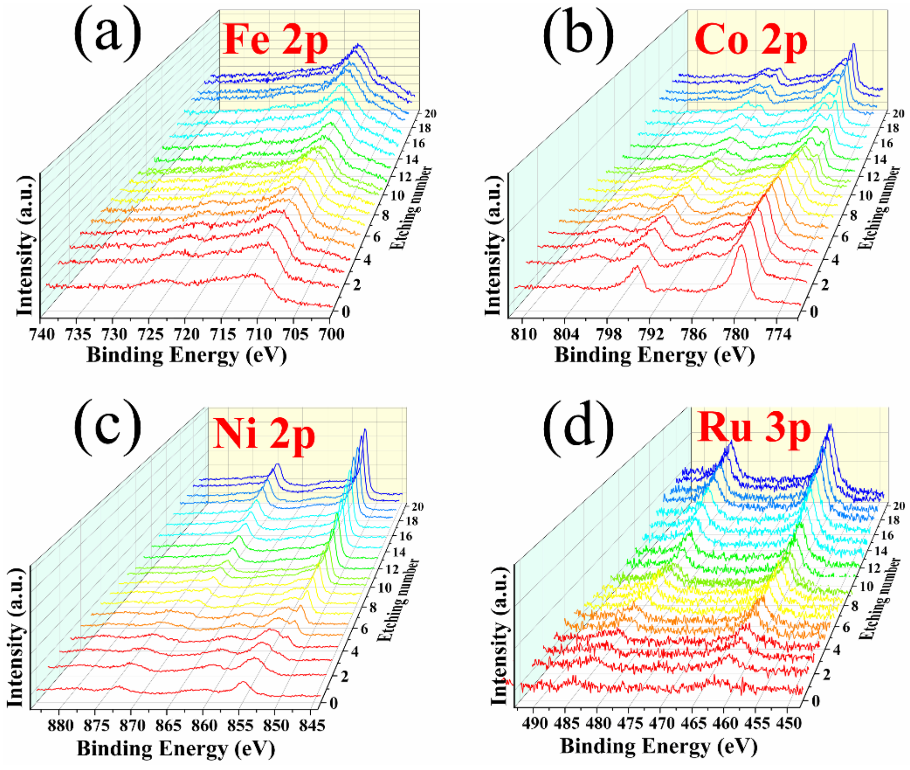

Figure S42 XPS compositional analysis of FeCoNiRu-450 nanoparticles after the OER stability test. (a-d) Fe 2p, Co 2p, Ni 2p, and Ru 3p spectra of FeCoNiRu-450 at different number of times of etching.

To evaluate the degree and depth of oxidation, the etching treatment of the surface of FeCoNiRu-450 electrocatalyst after the long OER was carried out using the same etching method as the long HER. Oxidation decays across the depth towards the matrix, also proving that the carbon layers are protective and can prevent the inner FeCoNiRu-450 nanoparticles from further oxidation. These XPS results are also consistent with the HRTEM analysis.

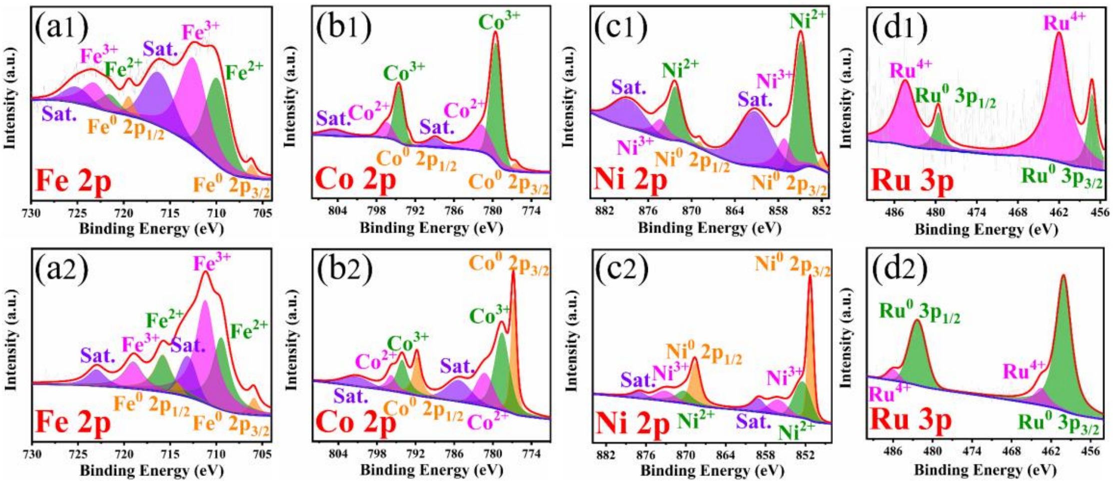

Figure S43 XPS compositional analysis of FeCoNiRu-450 nanoparticles after the OER stability test. High-resolution spectra of Fe, Co, Ni, and Ru before (a1-d1) and after (a2-d2) etching, respectively.

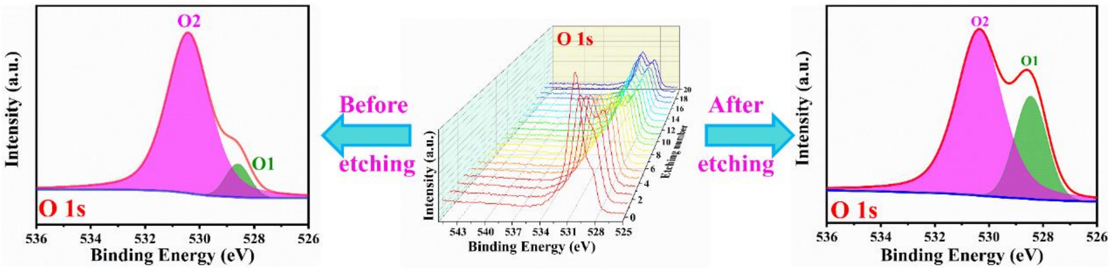

Figure S44 XPS compositional analysis of FeCoNiRu-450 nanoparticles after the OER stability test. O 1s spectra before and after etching process.

Two peaks of O1 and O2 are attributed to the lattice oxygen and surface-adsorbed oxygen-containing species (such as hydroxide or water).

Tabel S17 OH-adsorption free energy at each hollow sites of spinel oxide.

**Hollow sites (tet-oct-oct)**	**Adsorption energy (eV)**	**Hollow sites (tet-oct-oct)**	**Adsorption energy (eV)**
CoCoCo	-2.7464	CoNiCo	-2.7734
FeCoCo	-3.3320	CoNiRu	-2.8056
NiCoCo	-2.2642	CoRuCo	-2.7621
CoFeFe	-2.7574	FeFeCo	-3.3225
FeFeFe	-3.3402	FeFeNi	-3.3131
NiFeFe	-2.2690	FeFeRu	-3.3584
CoRuRu	-2.8198	FeNiCo	-3.3271
FeRuRu	-3.3747	FeNiRu	-3.3659
NiRuRu	-3.0268	FeRuCo	-3.3264
CoNiNi	-2.7436	NiFeCo	-2.2639
FeNiNi	-3.3282	NiFeNi	-2.2753
NiNiNi	-2.2321	NiFeRu	-3.0105
CoFeCo	-2.7609	NiNiCo	-2.2392
CoFeNi	-2.6310	NiNiRu	-2.2136
CoFeRu	-2.7788	NiRuCo	-2.3044

Reference

[1] A. C. Ferrari, D. M. Basko, *Nat. Nanotechnol.* **2013**, *8*, 235.

[2] Y. Pan, K. Sun, S. Liu, X. Cao, K. Wu, W.-C. Cheong, Z. Chen, Y. Wang, Y. Li, Y. Liu, *J. Am. Chem. Soc.* **2018**, *140*, 2610.

[3] W. Zhang, X. Yao, S. Zhou, X. Li, L. Li, Z. Yu, L. Gu, *Small* **2018**, *14*, 1800423.

[4] S. Gao, S. Hao, Z. Huang, Y. Yuan, S. Han, L. Lei, X. Zhang, R. Shahbazian-Yassar, J. Lu, *Nat. Commun.* **2020**, *11*, 2016.

[5] Z. Jia, T. Yang, L. Sun, Y. Zhao, W. Li, J. Luan, F. Lyu, L. Zhang, J. J. Kruzic, J. Kai, *Adv. Mater.* **2020**, *32*, 2000385.

[6] X. Zhao, Z. Xue, W. Chen, Y. Wang, T. Mu, *ChemSusChem* **2020**, *13*, 2038.

[7] P. Ma, M. Zhao, L. Zhang, H. Wang, J. Gu, Y. Sun, W. Ji, Z. Fu, *J. Mater.* **2020**, *6*, 736.

[8] G. Zhang, K. Ming, J. Kang, Q. Huang, Z. Zhang, X. Zheng, X. Bi, *Electrochim. Acta* **2018**, *279*, 19.

[9] W. Zhong, B. Xiao, Z. Lin, Z. Wang, L. Huang, S. Shen, Q. Zhang, L. Gu, *Adv. Mater.* **2021**, *33*, 2007894.

[10] Z. Wang, M. Li, J. Yu, X. Ge, Y. Liu, W. Wang, *Adv. Mater.* **2020**, *32*, 1906384.

[11] S. Fang, X. Zhu, X. Liu, J. Gu, W. Liu, D. Wang, W. Zhang, Y. Lin, J. Lu, S. Wei, *Nat. Commun.* **2020**, *11*, 1029.

[12] X. Zhang, Z. Luo, P. Yu, Y. Cai, Y. Du, D. Wu, S. Gao, C. Tan, Z. Li, M. Ren, *Nat. Catal.* **2018**, *1*, 460.

[13] Z. Zhao, H. Liu, W. Gao, W. Xue, Z. Liu, J. Huang, X. Pan, Y. Huang, *J. Am. Chem. Soc.* **2018**, *140*, 9046.

[14] H. Song, M. Wu, Z. Tang, J. S. Tse, B. Yang, S. Lu, *Angew. Chemie Int. Ed.* **2021**, *60*, 7234.

[15] M. M. Flores Espinosa, T. Cheng, M. Xu, L. Abatemarco, C. Choi, X. Pan, W. A. Goddard III, Z. Zhao, Y. Huang, *ACS Energy Lett.* **2020**, *5*, 3672.

[16] X. Zhang, F. Zhou, S. Zhang, Y. Liang, R. Wang, *Adv. Sci.* **2019**, *6*, 1900090.

[17] C. Cui, R. Cheng, H. Zhang, C. Zhang, Y. Ma, C. Shi, B. Fan, H. Wang, X. Wang, *Adv. Funct. Mater.* **2020**, *30*, 2000693.

[18] J. Wang, W. Fang, Y. Hu, Y. Zhang, J. Dang, Y. Wu, B. Chen, H. Zhao, Z. Li, *Appl. Catal. B Environ.* **2021**, *298*, 120490.

[19] T. Wang, H. Chen, Z. Yang, J. Liang, S. Dai, *J. Am. Chem. Soc.* **2020**, *142*, 4550.

[20] H. Li, H. Zhu, Q. Shen, S. Huang, S. Lu, P. Ma, W. Dong, M. Du, *Chem. Commun.* **2021**, *57,* 2637.

[21] T. X. Nguyen, Y. Liao, C. Lin, Y. Su, J. Ting, *Adv. Funct. Mater.* **2021**, *31,* 2101632.

[22] K. Huang, B. Zhang, J. Wu, T. Zhang, D. Peng, X. Cao, Z. Zhang, Z. Li, Y. Huang, *J. Mater. Chem. A* **2020**, *8*, 11938.

[23] G. Fang, J. Gao, J. Lv, H. Jia, H. Li, W. Liu, G. Xie, Z. Chen, Y. Huang, Q. Yuan, *Appl. Catal. B Environ.* **2020**, *268*, 118431.

[24] Z. Li, W. Niu, Z. Yang, N. Zaman, W. Samarakoon, M. Wang, A. Kara, M. Lucero, M. V Vyas, H. Cao, *Energy Environ. Sci.* **2020**, *13,* 884.

[25] X. Zheng, P. Cui, Y. Qian, G. Zhao, X. Zheng, X. Xu, Z. Cheng, Y. Liu, S. X. Dou, W. Sun, *Angew. Chemie* **2020**, *132*, 14641.

[26] Z. Zhang, X. Li, C. Zhong, N. Zhao, Y. Deng, X. Han, W. Hu, *Angew. Chemie Int. Ed.* **2020**, *59,* 7245.

[27] M. Wu, M. Cui, L. Wu, S. Hwang, C. Yang, Q. Xia, G. Zhong, H. Qiao, W. Gan, X. Wang, *Adv. Energy Mater.* **2020**, *10*, 2001119.

[28] D. Cao, H. Xu, D. Cheng, *Adv. Energy Mater.* **2020**, *10*, 1903038.

[29] A. Saad, D. Liu, Y. Wu, Z. Song, Y. Li, N. Tayyaba, K. Zong, P. Tsiakaras, X. Cai, *Appl. Catal. B Environ.* **2021**, *298*, 120529.

[30] S. Zhang, G. Zhan, X. Wang, S. Cao, Q. Yang, L. Yang, M. Li, J. Han, X. Zhu, H. Wang, *Appl. Catal. B Environ.* **2020**, *269*, 118782.

[31] J. Liu, J. Xiao, Z. Wang, H. Yuan, Z. Lu, B. Luo, E. Tian, G. I. N. Waterhouse, *ACS Catal.* **2021**, *11*, 5386.

[32] Y. Peng, Q. Liu, B. Lu, T. He, F. Nichols, X. Hu, T. Huang, G. Huang, L. Guzman, Y. Ping, *ACS Catal.* **2021**, *11*, 1179.

[33] X. Wu, B. Feng, W. Li, Y. Niu, Y. Yu, S. Lu, C. Zhong, P. Liu, Z. Tian, L. Chen, *Nano Energy* **2019**, *62*, 117.
